# Design and Synthesis of Hydroxyethylene-Based BACE-1 Inhibitors Incorporating Extended P1 Substituents

**DOI:** 10.2174/1874104501307010001

**Published:** 2013-03-08

**Authors:** Veronica Sandgren, Marcus Bäck, Ingemar Kvarnström, Anders Dahlgren

**Affiliations:** Department of Chemistry, Linköping University, S-581 83 Linköping, Sweden

**Keywords:** Alzheimer’s disease, BACE-1 inhibitors, extended P1 substituents, hydroxyethylene isosteres, P1´ modifications, S1-S3 pocket.

## Abstract

Novel BACE-1 inhibitors with a hydroxyethylene central core have been developed. Modified P1´ and extended P1 substituents were incorporated with the aim to explore potential interactions with the S1´ and the S1-S3 pocket, respectively, of BACE-1. Inhibitors were identified displaying IC_50_ values in the nanomolar range, *i.e.* 69 nM for the most potent compound. Possible inhibitor interactions with the enzyme are also discussed.

## INTRODUCTION

Alzheimer’s disease (AD) is a serious and fatal condition affecting tens of millions of individuals worldwide [1]. This form of dementia is primarily believed to be caused by the formation of insoluble polypeptides which form neurodegenerative plaques within the brain. One of the key enzymes involved in this formation is the human aspartic protease BACE-1 [2]. This knowledge together with the fact that no amyloid plaques are found in knock-out mice lacking BACE-1, while they seem to be vital and fertile, make inhibition of BACE-1 an interesting approach for targeting AD [3,4]. 

Earlier studies of aspartic proteases such as the malaria plasmepsins [5] and human renin [6] have shown that the S1 pocket of these enzymes can accommodate large P1 residues, many of which can reach into the S3 pocket. In a previous study we explored the possibility of extending deep into the BACE-1 S1 pocket by employing statine-based inhibitors with phenyloxymethyl residues in the P1 position Fig. (**1**) [7]. Some of these inhibitors displayed substantial activity against BACE-1 (12-40 nM), in the enzyme assay, but they were found to be inactive in a cell-based assay, likely due to poor permeability.

In the present work we have further investigated the possibility of improving cell permeability by reducing the polarity of the inhibitors. We also decided to focus on a hydroxyethylene (HE) central core in place of the statine-based isostere. An HE core with an *O*-methyl P1´ functionality was previously and successfully employed in our laboratories to synthesize potent BACE-1 inhibitors [8]. The reason for this change was that the carboxylic acid functionality of statine-based isosteres, which is a critical feature for high activity for this class, render inhibitors with poor cell-based permeability [7]. Further, the HE isostere contains one less amide linkage compared to the statine core, which decreases the peptidic character of these inhibitors. In addition, various substituents at the P1´ position have been evaluated.

Based on these observations, the synthesis of a series of hydroxyethylene-based inhibitors containing different P1´ residues and P1 substituents of variable sizes was undertaken. The main objective was to further investigate potential interactions with the S1-S3 binding pocket of BACE-1 Fig. (**1**).

## RESULTS AND DISCUSSION

### Chemistry 

A general structure of the inhibitors that were synthesized, along with the various R substituents used, is illustrated in Fig. (**2**). 

**Scheme 1:**
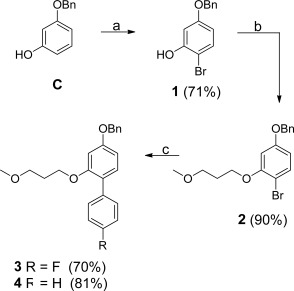
Reagents and conditions: (a) NBS, DCM, -15 °C; (b) 3-methoxy-1-propanol, PPh_3_, DIAD, THF, 0 °C to room temperature; (c) 4- fluorophenylboronic acid or phenylboronic acid, K_3_PO_4_, PEPPSI™-IPr catalyst, DMF, EtOH, H_2_O, 125 °C (microwave heating)

R^4^ amines **M**-**O **were synthesized from commercially available precursors using standard coupling and deprotection procedures (see the experimental section) and amine **P** was synthesized from commercially available precursors according to a three step protocol (including coupling, reduction and deprotection) (see the experimental section). The phenols **C** and **D** were commercially available. The P1 R^2^ residues **E **and **F **were synthesized from their corresponding benzyl ethers, as shown in Schemes **1** and **3**. An electrophilic aromatic substitution on 3-benzyloxy-phenol (**C**), utilizing *N*-bromosuccinimide (NBS) in DCM at –15 °C, produced aryl halide **1** in 71% yield (Scheme **1**) [9]. Compound **1 **was subsequently subjected to Mitsunobu-like conditions using 3-methoxy-1-propanol, PPh_3_, and DIAD in THF to give compound **2 **in 90% yield. We then applied microwave-assisted palladium coupling conditions, which in this case involved treatment of **2** with 4-fluorophenylboronic acid or phenylboronic acid, K_3_PO_4_, and PEPPSI^™^-IPr catalyst in EtOH, H_2_O, and DMF. Microwave irradiation at 125 °C for 15 min produced biphenyl derivatives **3 **and** 4 **in 70% and 81% yield, respectively. The corresponding benzyl ether of compound **G **was synthesized from **C** by applying the same conditions utilized to prepare compound **2**, and compound **H **was obtained from commercially available 4-bromophenol using the same Suzuki protocol employed to synthesize compound** 3**. Compounds **A** and** B** were prepared according to reported procedures [10, 11].

**Scheme 2:**
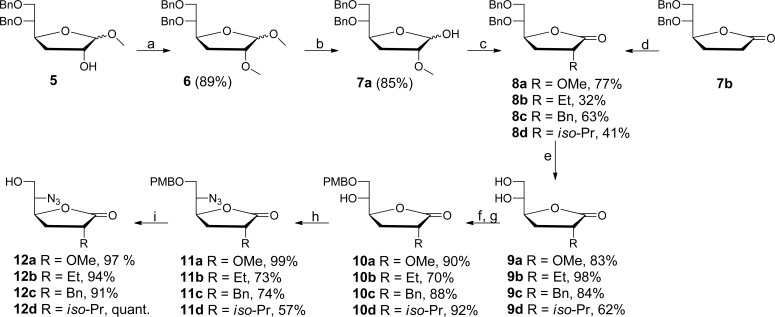
Synthesis of compounds **12a-d**. Compound **7a** was used for the synthesis of **8a**, while compound **7b** was employed for the synthesis of **8b-d**. Reagents and conditions: (a) MeI, Ag_2_O, DMF; (b) H_2_SO_4_, dioxane; (c) PDC, 4 Å molecular sieves; (d) (i) LDA, tripyrrolidinophosphine oxide, EtBr or 2-iodopropane, THF (dry), -68 °C or (ii) LDA, BnBr, THF (dry), -78 °C; (e) (i) H_2_/Pd(OH)_2_-C, AcOH, EtOH or (ii) H2/Pd-C, EtOH; (f) Bu_2_SnO, toluene, reflux; (g) tetrabutylammonium bromide, 4-OMeBnBr, toluene, 90 °C; (h) PPh_3_, DIAD, DPPA, THF (dry); (i) DDQ, H_2_O, DCM.

The synthesis of compounds **12a-d** is depicted in Scheme **2**. 1,2:5,6-Di-*O*-isopropylidene-a-D-glucofuranose was used as a precursor from which compound **5 **was synthesized in 49% yield over five steps using a method described in the literature [12-15]. Treatment of **5 **with MeI and Ag_2_O in DMF furnished the methyl ether **6 **in 89% yield. Hydrolysis of **6** using H_2_SO_4 _in dioxane gave hemiacetal **7a** in 85% yield, and the subsequent oxidation with PDC in DCM produced lactone **8a **in 77% yield [8]. Lactone **7b **was synthesized according to a literature procedure [13] and was used as a precursor for synthesizing compounds **8b-d**. Stereoselective alkylation at the *C2* position of **7b** was achieved using LDA, tripyrrolidinophosphine oxide, and EtBr or 2-iodopropane in THF at -68 °C to furnish the alkylated lactones **8b **and **8d** in 32% and 41% yield, respectively. Compound **8c** was produced in 63% yield from **7b** using a similar procedure with LDA and BnBr in THF at -78 °C. Next, compounds **8a-d** were exposed to catalytic hydrogenolysis to prepare the diols **9a-d **in 62-98% yield. Selective primary benzylation [16] was performed by converting **9a-d** into their corresponding tin acetals by reacting the diols with Bu_2_SnO in refluxing toluene, followed by treatment with tetrabutylammonium bromide and 4-methoxybenzyl bromide to furnish the monobenzyl ethers **10a-d** in 70-92% yield over two steps. Employing a Mitsunobu-like protocol with PPh_3_, DIAD, and DPPA in dry THF provided azides **11a-d** in 57-99% yield. Finally, an oxidative removal of the *p*-methoxybenzyl group using 2,3-dichloro-5,6-dicyano benzoquinone (DDQ) in H_2_O and DCM, delivered compounds **12a-d** in 91-100% yield [17].

**Scheme 3:**
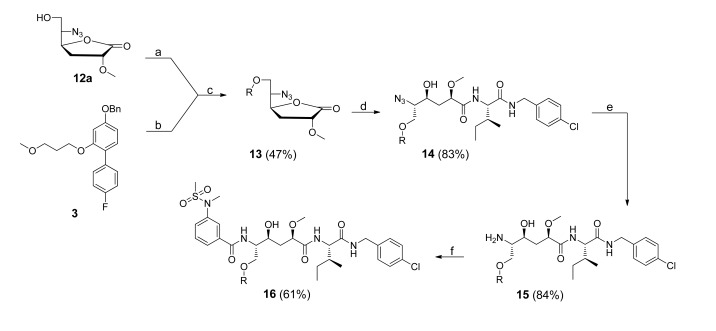
Reagents and conditions: (a) triflic anhydride, pyridine (dry), DCM (dry); (b), H_2_/Pd-C, EtOH; (c) Cs_2_CO_3_, 4 Å molecular sieves, DCM (dry), 40 °C; (d) **O**, 2-hydroxypyridine, DIPEA, DMF, 75 °C; (e) PPh_3_, MeOH, H_2_O; (f) **A**, DIPEA, HATU, DMF.

In Scheme **3** the synthesis of target compounds **15 **and **16** is presented. Initially, compound **3 **was converted to the corresponding phenol by catalytic hydrogenolysis using H_2_/Pd-C in ethanol. Alcohol **12a** was then reacted with Tf_2_O and pyridine in DCM to yield the corresponding triflate. The phenol and triflate were used without further purification in a substitution reaction, using Cs_2_CO_3_ in DCM containing 4 Å molecular sieves, to give compound **13** in 47% yield. It should be noted that we first applied Mitsunobu conditions in an attempt to produce **13**. However, the use of this protocol led to significant elimination problems whereas the former approach described above reduced this side reaction considerably. This could be achieved by using fresh Cs_2_CO_3 _in dry DCM, and by letting the reaction proceed for a short time at 40 °C under N_2_ atmosphere. The lactone **13** was opened using amine **O **and****2-hydroxypyridine in DIPEA and DMF at 75 °C, which gave **14** in 83% yield. Reduction using PPh_3_ in H_2_O and methanol afforded target compound **15** in 84% yield. Amine **15** was coupled with carboxylic acid **A **using HATU and DIPEA in DMF to provide target compound **16** (61%). It should be noted that the steps described in Scheme **3** are a representative final route for synthesizing the target compounds. All inhibitors were prepared following this general procedure with some variations, *i.e.* different phenols were used in step c, opening of the lactone was performed with amines **M**-**P** and the final peptide coupling step was only performed in the synthesis of final products **16**, **18**, **19**, **24**, and **25** (see the experimental section). Attempts to co-crystallize inhibitor **15** with BACE-1 were performed, but these were not successful.

### Structure activity relationships

The target compounds are summarized in Table **1**. They were synthesized from compounds **12a-d** according to Scheme **3** using appropriate R substituents****shown in Fig. (**2**). Enzyme activities were measured against BACE-1, and the IC_50_ values are presented in Table **1**. In addition, percent inhibition in a cell-based assay was determined at the concentration 1 µM for the four most potent inhibitors (compounds **25**, **27**, **28**, and **30**).

 Initially, *m*-benzyloxy insertion in the P1 position of the general structure outlined in Table **1** gave the inactive amine **17 **and the modestly active sulfonamide **18 **(IC_50_ values > 10 and 2.2 μM, respectively). The P1 *p*-benzyloxy-modified sulfonamide **19** proved to be nearly equipotent (IC_50_ 4.6 μM) to the corresponding P1 *m*-benzyloxy compound **18**. Nonetheless, the results for **18 **and **19** were promising and indicated that more advanced P1 substituents could provide inhibitors with increased activity. Further development led to BACE-1 inhibitors **20 **and** 15 **containing the P1 R^2^ substituent **E **and P2´-P3´ R^4 ^substituents **N** and **O **Fig. (**2**) with a *p*-chloro substituent in the P3´ part of the molecule. Compound **15 **has an isoleucine in the P2´ position and an IC_50_ value of 1.2 μM, making it slightly more active than the corresponding P2´ valine-containing inhibitor **20 **(IC_50 _2.7 μM). In addition, the fluorine substituent on the P1 moiety seems to contribute somewhat to the activity, which can be tentatively concluded when looking at compound **21** (IC_50_ 2.5 μM), lacking the fluorine. The activity of this class of inhibitors most likely depends on interactions from both the *para *and the *meta* groups on the P1 substituent, as observed for targets **22 **and **23 **(IC_50_ values > 10 μM) lacking the *para* and the *meta *substituent, respectively. Notably, **15** is a moderately more potent inhibitor than **18**, even though it does not contain a P2 substituent. This can probably be explained by an interaction effect of the more advanced P1 substituent. Unfortunately, introduction of the P2 sulfonamide substituent **A** into **15 **and **21 **rendered compounds highly hydrophobic in character, which caused precipitation problems during the activity measurements and thus no inhibition data were obtained for **16 **and **24**. Compound **25 **contains the larger P2-P3 portion **B** and has a notable IC_50_ value of 69 nM, which is very intriguing, since it might indicate that the active site possibly can accommodate such a large compound having both the P3 portion and the sizable P1 residue. Further exploration of the P1´ and P3´ positions were done to see if a more drug-like compound could be found, with the P2-P3 substituent excluded. Modification in the P1´ position resulted in compound **26** which proved to be almost equipotent with compound **15**. Also, compound **29** proved to be roughly equally potent as **15**. Compound **27** has a benzyl group in the P1´ position and an IC_50_ value of 0.47 μM, making it somewhat more active than inhibitor **15**. To investigate if the amide-bond between P2´ and P3´ contributes to biological activity, compounds **28** and **30** were synthesized. Interestingly, **28** and **30** have IC_50_ values of 0.30 and 0.54 μM, respectively, which are equal to the activities seen for compounds **27** and **29**, indicating that the P2´-P3´ amide portion is non-critical for BACE-1 activity in this series of inhibitors.

 Even though we were able to exclude the P2-P3 portion of the inhibitors and still maintain promising enzyme activities in the nanomolar range, the size and the overall polarity of the compounds unfortunately resulted in poor activities in the cell-based assay with 3%, 19%, 14%, and 28% inhibition of BACE-1 at the concentration 1 μM for the four most active inhibitors **25**, **27**, **28**, and **30**, respectively. The fact that compound **25** shows such a low inhibition as 3% can most likely be explained by its size and peptide-like structure. As expected, decreased size and less peptidic character lead to increased inhibition in cells. 

## CONCLUSION

We have synthesized a series of BACE-1 inhibitors based on a hydroxyethylene core. Although our focus has been on the P1 part of the target molecules, other positions of the inhibitors have also been varied and studied. Enzyme measurements (to give IC_50_ values) have been performed on the inhibitors and percent inhibition in a cell-based assay was measured for the four most active inhibitors. Various side chain substituents have been employed and the best inhibitor (target **25**) displayed an enzyme IC_50_ of 69 nM. Furthermore, target molecules **27**-**30** all had IC_50 _values in the nanomolar range. Interestingly, though they lack a P2 substituent, they still performed well potency-wise. Moreover, compounds **28** and **30 **possess only one amide bond, which make them more drug-like when compared with other published inhibitors [18]. The cell-based assays revealed that, as could be foreseen, smaller molecular size and less peptidic character led to increased activity in this regard. Attempts to co-crystallize inhibitor **15** with BACE-1 were done, but were unfortunately unsuccessful. X-ray data could have given us valuable information about interactions between our compounds and BACE-1, which hopefully would have provided us with new ideas on further optimizations. Further research should therefore be aimed at obtaining X-ray structures showing the interactions of our best inhibitors in the active site of BACE-1. 

## EXPERIMENTAL SECTION

### General 

NMR-spectra were recorded on a Varian 300 MHz instrument using CDCl_3_ and CD_3_OD as solvents. TLC was carried out on Merck precoated 60 F_254_ plates using UV-light and charring with ethanol/sulfuric acid/*p*-anisaldehyde/acetic acid 90:3:2:1 or a solution of 0.5% ninhydrin in ethanol for visualization. Flash column chromatography was performed using silica gel 60 (0.040-0.063 mm, Merck). Organic phases were dried over anhydrous magnesium sulfate. Concentrations were performed under diminished pressure at a bath temperature of 40 °C. Optical rotations were measured using a Perkin-Elmer 141 polarimeter.

Gradient LC-MS was performed on a Gilson system (column: Phenomenex C-18 250 x 15 mm, 10 µm and Phenomenex C-18 150 x 4.6 mm, 5 µm for preparative and analytical runs, respectively; pump: Gilson gradient pump 322; UV/VIS-detector: Gilson 152; MS detector: Thermo Finnigan Surveyor MSQ; Gilson Fraction Collector FC204) using methanol with 0.1% formic acid and deionized water with 0.1% formic acid as mobile phases. High resolution mass spectra (HRMS) were recorded on a Waters Synapt HDMS instrument equipped with an electrospray interface.

### BACE-1 Enzyme Assay 

The BACE-1 assay was performed as previously described [7]. The inhibition of BACE-1 was determined in a homogeneous time resolved fluorescence (TRF) assay (True Point kit, Perkin-Elmer). The assay buffer contained sodium acetate, CHAPS, Triton-X 100, and EDTA, pH 4.5. The substrate used was the Swedish mutant sequence Eu-EVNLDAEFK-Quencher.

### BACE-1 Cell Assay

The cell-based assay was performed as previously described [13]. Human embryonic kidney 293 cells (HEK-293) stably expressing Swedish mutant APP (swAPP751) were cultured in DMEM supplemented with 10% FCS, PEST (50 U penicillin/50 mg/mL streptomycin) and 200 mg Hygromycin/mL at 37 °C, 5% CO_2_. After 24 h cell culture media was harvested and analysed by Ab 1–40 ELISA according to the manufacturer instruction (The Genetics Company, Schwitzerland). The results were treated with the curve fitting package in GraphPad Prism 5.

### LC-MS Purity Measurements

#### Chromatography System A

Peaks were detected at 254 nm. Column: Phenomenex C-18 150 x 4.6 mm, 5 µm. Gradient: methanol 40-100% over 10 min at 1 mL/min followed by 100% methanol for 5 min. Formic acid (0.1% v/v) was added to all solvents .

#### Chromatography System B

Peaks were detected at 254 nm. Column: XBridge™ C-18, 4.6 x 50 mm, 2.5 µm. Mobile phase A: 10 mM NH_4_OAc in 95:5 H_2_O/ACN; mobile phase B: 10 mM NH_4_OAc in 90:10 ACN/H_2_O. Gradient: 30-100% B over 6 min at 1 mL/min followed by 100% B for 3 min. 

### Synthetic Protocols (used in the Synthesis of Building Blocks M-O)

#### Peptide Coupling

 To a cooled (0 °C) solution of the acid (4.95 mmol) in DMF (20 mL) were added the amine (5.44 mmol), DIPEA (14.84 mmol) and HATU (5.94 mmol). The solution was stirred at 0 °C for 0.5 h and for an additional 2 h at room temperature. EtOAc and brine were added and the organic phase was separated and washed once with brine, dried and concentrated. The crude peptide mixture was purified by flash column chromatography.

#### Boc Deprotection

To a solution of the protected compound (0.167 mmol) and triethylsilane (0.419 mmol) in DCM (3 mL) was added TFA (1 mL). The solution was stirred for 2 h at room temperature and then concentrated and co-evaporated with toluene.

### Synthesis of Building block P.

#### Tert-Butyl ((2S,3S)-1-((4-chlorophenyl)amino)-3-methyl-1-oxopentan-2-yl)carbamate

To a solution of Boc-Ile-OH (1.00 g, 4.32 mmol) in THF (10 mL) cooled to -20˚C under N_2_-atmosphere *N*-methyl morpholine (500 μL, 4.55 mmol) was added and the mixture was stirred for 10 minutes. Isobutylchloroformate (590 μL, 4.55 mmol) was added while keeping the temperature below -14˚C. After 20 minutes 4-chloroaniline (520 μL, 4.78 mmol) was added dropwise while keeping the temperature below -20˚C. The reaction mixture was allowed to attain room temperature over five hours before being diluted with toluene and washed with HCl (aq., 2M) and H_2_O. The organic phase was dried, filtered and evaporated. Purification using flash column chromatography (toluene/EtOAc 19:1) provided the product (1.06 g, 72%) as a white solid. ^1^H NMR (300 MHz, CDCl_3_): δ 0.90 (t, *J *= 7.4 Hz, 3H), 1.00 (d, *J *= 6.9 Hz, 3H), 1.12-1.28 (m, 1H), 1.42 (s, 9H), 1.55-1.70 (m, 1H), 1.90-2.00 (m, 1H), 4.10 (t, *J *= 7.9 Hz, 1H), 5.30 (d, *J *= 6.9 Hz, 1H), 7.15 (d, *J *= 8.5 Hz, 2H), 7.39 (d, *J *= 8.8 Hz, 2H), 8.64 (s, 1H); ^13^C NMR (75.5 MHz, CDCl_3_): δ 11.2, 15.7, 25.1, 25.1, 28.5, 37.0, 60.2, 80.7, 121.2, 128.9, 129.3, 136.4, 156.6, 170.8.

#### Tert-Butyl ((2S,3S)-1-((4-chlorophenyl)amino)-3-methylpentan-2-yl)carbamate (Boc-protected P)

Compound tert-Butyl ((2S,3S)-1-((4-chlorophenyl)amino)-3-methyl-1-oxopentan-2-yl)carbamate (1.00 g, 2.92 mmol) was dissolved in THF/toluene (1:2, 3.3 mL) and cooled to 0˚C under N_2_-atmosphere. Red-Al (65 wt. % in toluene, 4.70 mL, 15.41 mmol) was added dropwise and the solution was heated to 50˚C. After 24 hours the reaction mixture was cooled to 0˚C and quenched with NaOH (aq., 2 M, 16 mL) during 40 minutes before being diluted with toluene. The organic phase was washed with NaOH (aq., 2 M) and H_2_O and was then dried, filtered and concentrated. The crude product was purified using flash column chromatography (toluene/EtOAc 9:1) to furnish the product (0.82 g, 85%) as a white solid. ^1^H NMR (300 MHz, CDCl_3_): δ 0.88-1.00 (m, 6H), 1.10-1.24 (m, 1H), 1.45 (s, 9H), 1.46-1.64 (m, 2H), 2.85-3.04 (m, 1H), 3.18-3.24 (m, 1H), 3.75 (bs, 1H), 4.14 (bs, 1H), 4.52 (bs, 1H), 6.42-6.51 (m, 2H), 7.03-7.10 (m, 2H); ^13^C NMR (75.5 MHz, CDCl_3_): δ 11.6, 15.5, 25.4, 28.4, 37.4, 46.7, 54.7, 79.6, 113.7, 121.6, 129.0, 147.2, 156.7.

#### (2S,3S)-N^1^-(4-chlorophenyl)-3-methylpentane-1,2-diamine (P)

Boc-protected **P** (145 mg, 0.44 mmol) was dissolved in dioxane (9 mL) and HCl (conc., 3 mL) was added. The mixture was stirred at room temperature for 15 min and co-evaporated with toluene. Purification using flash column chromatography (MeOH/EtOAc 1:9 + 1% TEA) gave the amine **P** (98 mg, 98%) as a colorless oil. ^1^H NMR (300 MHz, CDCl_3_): δ 0.91-0.96 (m, 6H), 1.16-1.26 (m, 1H), 1.38-1.47 (m, 1H), 1.49-1.60 (m, 1H), 2.75-2.85 (m, 2H), 3.14-3.24 (m, 1H), 6.54 (d, *J* = 9.0 Hz, 2H), 7.10 (d, *J* = 9.0 Hz, 2H); ^13^C NMR (75.5 MHz, CDCl_3_): δ 11.7, 15.3, 25.3, 40.0, 47.6, 55.1, 114.2, 121.9, 129.1, 147.5.

### Synthetic Procedures

#### 5-Benzyloxy-2-bromo-phenol (1)

A mixture of 3-benzyloxyphenol (**C**) (122 mg, 0.609 mmol) in dry DCM (4 mL) was cooled to -15 °C and NBS (109 mg, 0.609 mmol) was added. The solution was stirred at -10 °C for 40 min and for an additional 20 min at room temperature. Concentration and flash column chromatography (DCM/hexanes 7:3) gave **1** (121 mg, 71%) as a colorless oil. [α]_D_^22^ -10 (c 0.1, MeOH); ^1^H NMR (300 MHz, CDCl3) δ 4.97 (s, 2H), 5.53 (bs, 1H), 6.41 (dd, *J* = 2.7, 8.8 Hz, 1H), 6.65 (d, *J* = 2.8 Hz, 1H), 7.28 (d, *J* = 8.8 Hz, 1H), 7.31-7.41 (m, 5H); ^13^C NMR (75.5 MHz, CDCl3) δ 70.3, 101.3, 102.8, 109.3, 127.5, 128.2, 128.7, 132.1, 136.5, 153.0, 159.7. MS (M+H)^+^ calcd: 279.0; found: 279.1.

#### 4-Benzyloxy-1-bromo-2-(3-methoxy-propoxy)-benzene (2)

To a cooled (0 °C) solution of **1** (680 mg, 2.44 mmol), 3-methoxy-1-propanol (467 μL, 4.87 mmol) and PPh_3_ (1.28 g, 4.88 mmol) in dry THF (60 mL) DIAD (960 μL, 4.88 mmol) was added. The mixture was then allowed to attain room temperature overnight. Concentration and flash column chromatography (toluene) provided **2** (770 mg, 90%) as a colorless oil. [α]_D_^22^ -16 (*c* 0.1, MeOH); ^1^H NMR (300 MHz, CDCl_3_) δ 2.02-2.12 (m, 2H), 3.35 (s, 3H), 3.59 (t, *J *= 6.1 Hz, 2H), 4.06 (t, *J *= 6.2 Hz, 2H), 5.00 (s, 2H), 6.44 (dd, *J *= 2.8, 8.5 Hz, 1H), 6.57 (d, *J *= 2.8 Hz, 1H), 7.28-7.43 (m, 6H); ^13^C NMR (75.5 MHz, CDCl_3_) δ 29.5, 58.9, 65.9, 69.1, 70.4, 101.9, 103.3, 107.1, 127.6, 128.2, 128.7, 133.1, 136.6, 156.5, 159.3. MS calcd (M+Na)^+^: 373.0; found 373.5. 

#### 4-Benzyloxy-4'-fluoro-2-(3-methoxy-propoxy)-biphenyl (3)

A microwave process vial was charged with **2** (100 mg, 0.285 mmol), 4-fluorophenylboronic acid (80 mg, 0.572 mmol), K_3_PO_4 _(150 mg, 0.71 mmol), PEPPSI^™^-IPr catalyst (10 mg, 0.015 mmol), DMF (2.8 mL), EtOH (1.2 mL), and H_2_O (0.8 mL). The vial was capped with a Teflon septum and irradiated with microwaves to 125 °C for 15 min. Cooling followed by concentration and flash column chromatography (toluene) gave **3** (73 mg, 70%) as a colorless oil. [α]_D_^22^ +9.0 (*c* 0.1, MeOH); ^1^H NMR (300 MHz, CDCl_3_) δ 1.88-1.99 (m, 2H), 3.27 (s, 3H), 3.41 t, *J *= 6.0 Hz, 2H), 4.00 t, *J *= 6.0 Hz, 2H), 5.04 (s, 2H), 6.59 (dd, *J *= 2.5, 8.2 Hz, 1H), 6.64 (d, *J *= 2.5 Hz, 1H), 7.03 (app. t, *J*_HH_**= *J*_HF_ = 8.8 Hz, 2H), 7.16 (d, *J *= 8.2 Hz, 1H), 7.27-7.48 (m, 7H); ^13^C NMR (75.5 MHz, CDCl_3_) δ 29.5, 58.7, 65.2, 69.2, 70.2, 100.8, 105.9, 114.7 (d, *J*_CF_ = 21.2 Hz, 2C), 123.0, 127.6, 128.1, 128.7, 131.0 (d, *J*_CF_ = 8.0 Hz, 2C), 131.1, 134.4, 136.9, 156.7, 159.5, 161.7 (d, *J*_CF_ = 245.1 Hz). MS calcd (M+H)^+^: 367.2; found 367.0. 

#### 4-Benzyloxy-2-(3-methoxy-propoxy)-biphenyl (4)

Compound **4** was synthesized from **2** in 81% yield (colorless oil) according to the method for synthesizing compound **3 **using phenylboronic acid instead of 4-fluorophenylboronic acid. [α]_D_^22^ +2.0 (*c* 0.1, MeOH); ^1^H NMR (300 MHz, CDCl_3_) δ 1.89-1.99 (m, 2H), 3.27 (s, 3H), 3.42 (t, *J *= 6.2 Hz, 2H), 4.00 (t, *J *= 6.2 Hz, 2H), 5.05 (s, 2H), 6.61 (dd, *J *= 2.3, 8.4 Hz, 1H), 6.65 (d, *J *= 2.5 Hz, 1H), 7.19-7.29 (m, 2H), 7.30-7.46 (m, 7H), 7.47-7.52 (m, 2H); ^13^C NMR (75.5 MHz, CDCl_3_) δ 29.5, 58.7, 65.2, 69.3, 70.2, 100.8, 105.9, 124.0, 126.4, 127.6, 127.9, 128.1, 128.7, 129.6, 131.3, 137.0, 138.5, 156.8, 159.5. MS calcd (M+H)^+^: 349.2; found 349.0. 

#### 1-Benzyloxy-3-(3-methoxy-propoxy)-benzene (benzylated G)

 This compound was synthesized in 86% yield (colorless oil) from 3-benzyloxyphenol (**C**) according to the preparation method for **2**. ^1^H NMR (300 MHz, CDCl_3_) δ 1.97-2.07 (m, 2H), 3.34 (m, 3H), 3.53 (t, *J* = 6.2 Hz, 2H), 4.02 (t, *J* = 6.2 Hz, 2H), 5.02 (s, 2H), 6.49-6.59 (m, 3H), 7.11-7.19 (m, 2H), 7.29-7.45 (m, 4H); ^13^C NMR (75.5 MHz, CDCl_3_) δ 29.7, 58.8, 65.0, 69.4, 70.1, 102.0, 107.2, 107.3, 127.6, 128.0, 128.7, 130.0, 137.2, 160.2, 160.4.

#### 4'-Fluoro-biphenyl-4-ol (H)

Compound **H** was synthesized in 96% yield (colorless solid) from 4-bromophenol according to the preparation method for **3**. ^1^H NMR (300 MHz, CD_3_OD) δ 4.92 (bs, 1H), 6.84 (d, *J* = 8.8 Hz, 2H), 7.03 (app. t, *J*_HH_= *J*_HF_ = 8.9 Hz, 2H), 7.34 (d, *J* = 8.8 Hz, 2H), 7.44 (dd, *J *= 5.4, 8.9 Hz, 2H); ^13^C NMR (75.5 MHz, CD_3_OD) δ 116.2 (d, *J*_CF_ = 21.5 Hz, 2C), 116.6, 128.9, 129.0 (d, *J*_CF_ = 8.0 Hz, 2C), 132.8, 138.6, 158.0, 163.2 (d, *J*_CF_ = 243.9 Hz).

#### 3-(Methanesulfonyl-methyl-amino)-benzoic acid (A)

 Commercially available 3-amino-benzoic acid methyl ester was used as starting material. The mesylation, methylation and ester hydrolysis steps were performed according to reference [10].

#### 5-(Methanesulfonyl-methyl-amino)-N-((R)-1-phenyl-ethyl)-isophthalamic acid (B)

Synthesized according to reference [11].

#### Methyl 5,6-di-O-benzyl-3-deoxy-D-glucofuranoside (5)

Compound **5** was synthesized in 49% total yield over five steps according to references [12-15]. Analytical data was in accordance with reference [13].

#### Methyl 5,6-di-O-benzyl-3-deoxy-2-O-methyl-D-glucofuranoside (6)

To a solution of **5** (3.52 g, 9.82 mmol) in DMF (40 mL) MeI (6.10 mL, 98.0 mmol) and Ag_2_O (4.55 g, 19.6 mmol) were added. The mixture was stirred at room temperature overnight, filtered and concentrated. Flash column chromatography (toluene/EtOAc 15:1) furnished **6** (3.26 g, 89%) as a colorless oil. [α]_D_^22^ -60 (*c* 0.1, MeOH); ^1^H NMR (300 MHz, CDCl_3_) δ 2.03-2.20 (m, 2H), 3.35 (s, 3H), 3.37 (s, 3H), 3.63-3.73 (m, 2H), 3.79-3.88 (m, 2H), 4.36-4.46 (m, 1H), 4.59 (d, *J* = 12.1 Hz, 1H), 4.64 (d, *J* = 12.1 Hz, 1H), 4.69 (d, *J* = 11.5 Hz, 1H), 4.84 (d, *J* = 11.5 Hz, 1H), 4.92 (s, 1H), 7.26-7.44 (m, 10H); ^13^C NMR (75.5 MHz, CDCl_3_) δ 32.1, 54.7, 56.8, 71.1, 73.0, 73.4, 79.1, 81.4, 85.0, 107.1, 127.5, 127.5, 127.6, 127.8, 128.3, 128.4, 138.5, 138.8. 

#### 5,6-Di-O-benzyl-3-deoxy-2-O-methyl-D-glucofuranoside (7a)

Compound **6** (2.31 g, 6.20 mmol) was dissolved in dioxane (65 mL) and 0.5 M H_2_SO_4 _(58 mL) was added. The solution was refluxed overnight and neutralized with saturated aqueous NaHCO_3_. The dioxane was evaporated and the residue was dissolved in DCM. Washing with H_2_O, drying, filtration, concentration and flash column chromatography (toluene/EtOAc 15:1 → toluene/EtOAc 6:1) gave **7a** (1.89 g, 85%) as a lightly yellowish oil. [α]_D_^22^ -19 (*c* 0.1, MeOH); ^1^H NMR (300 MHz, CDCl_3_) δ 1.86-2.02 (m, 1H), 2.05-2.20 (m, 1H), 3.34 (s, 3H), 3.40 (s, 1H), 3.58 (d, *J* = 5.2 Hz, 2H), 3.69 (d, *J* = 4.9 Hz, 1H), 3.85-3.92 (s, 1H), 4.44-4.51 (m, 1H), 4.53 (d, *J* = 2.2 Hz, 2H), 4.63 (d, *J* = 11.1 Hz, 1H), 4.79 (d, *J* = 11.1 Hz, 1H), 5.23 (d, *J* = 8.2 Hz, 1H), 7.26-7.39 (m, 10H); ^13^C NMR (75.5 MHz, CDCl_3_) δ 28.6, 57.1, 70.1, 73.5, 73.8, 79.1, 79.9, 86.3, 100.0, 127.8, 128.0, 128.2, 128.4, 128.7, 128.7, 137.8, 138.1.

#### (3R,5S)-5-((R)-1,2-Bis(benzyloxy)ethyl)-3-methoxy-dihydro-furan-2-one (8a)>

To a mixture of **7a** (1.84 g, 5.13 mmol) in DCM (30 mL) powdered 4 Å molecular sieves and PDC (3.86 g, 10.26 mmol) were added and the mixture was stirred at room temperature overnight. Filtration, concentration and flash column chromatography (toluene/EtOAc 18:1) provided **8a** (1.40 g, 77%) as a colorless oil. [α]_D_^22^ +7.0 (*c* 0.1, MeOH); ^1^H NMR (300 MHz, CDCl_3_) δ 2.12 (ddd, *J *= 7.0, 8.2, 13.5 Hz, 1H), 2.55 (ddd, *J* = 3.6, 8.2, 13.5 Hz, 1H), 3.47-3.61 (m, 2H), 3.51 (s, overlapped, 3H), 3.87 (ddd, *J* = 3.0, 5.1, 8.1 Hz, 1H), 4.10 (dd, *J* = 6.9, 8.2 Hz, 1H), 4.49 (d, *J* = 12.1 Hz, 1H), 4.54 (d, *J* = 12.1 Hz, 1H), 4.57, (d, *J* = 11.5 Hz, 1H), 4.65 d, *J* =11.5 Hz, 1H), 4.75 (ddd, *J* = 3.0, 3.7, 8.4 Hz, 1H), 7.21-7.40 (m, 10H); ^13^C NMR (75.5 MHz, CDCl_3_) δ 29.7, 58.3, 68.8, 73.7, 75.6, 78.1, 78.2, 127.8, 128.1, 128.2, 128.2, 128.6, 137.7, 175.0. MS calcd (M+H)^+^: 357.1; found 356.5. 

#### (3R,5S)-5-((R)-1,2-Bis-benzyloxy-ethyl)-3-ethyl-dihydro-furan-2-one (8b)

A solution of the lactone **7b** (299 mg, 0.92 mmol) in dry THF (3 mL) was added to a 2 M solution of LDA in THF (0.9 mL, 1.8 mmol) at -68 ºC under N_2_. The reaction mixture was then treated with tripyrrolidinophosphine oxide (1.4 mL, 6.1 mmol) and EtBr (300 µL, 4.0 mmol). Stirring at -60 ºC was continued for 1 h and the reaction mixture was quenched with aq. NH_4_Cl. The phases were separated and the aqueous phase was extracted twice with ethyl acetate. The combined organic extracts were dried, filtered and concentrated. The residue was purified using flash column chromatography (toluene/EtOAc 18:1 → toluene/EtOAc 9:1) which provided **8b** (104 mg, 32%) as a colorless oil. ^1^H NMR (300 MHz, CDCl_3_): δ 0.96 (t, *J *= 7.5 Hz, 3H), 1.40-1.55 (m, 1H), 1.76-1.93 (m, 2H), 2.36-2.45 (m, 1H), 2.53-2.64 (m, 1H), 3.53-3.63 (m, 2H), 3.81-3.86 (m, 1H), 4.48-4.70 (m, 5H), 7.26-7.39 (m, 10H); ^13^C NMR (75.5 MHz, CDCl_3_): δ 11.7, 24.5, 28.1, 40.8, 69.1, 73.6, 73.7, 78.0, 78.6, 127.8, 128.0, 128.1, 128.2, 128.6, 128.7, 137.9, 140.0, 179.7. MS calcd (M+H)^+^: 355.2; found 354.6.

#### (3R,5S)-3-Benzyl-5-((R)-1,2-bis-benzyloxy-ethyl)-dihydro-furan-2-one (8c)

The lactone **7b **(155 mg, 0.48 mmol) was dissolved in dry THF (2.5 mL) and cooled to -78 ºC under N_2_-atm. LDA (2M, 260 µL, 0.52 mmol) was added dropwise. After 15 min the temperature was raised to -50 ºC and benzyl bromide (240 µL, 2.0 mmol) was added dropwise. After 2 h the reaction mixture was allowed to attain room temperature and quenched with saturated NH_4_Cl (aq). The mixture was extracted with DCM three times. The combined organic layers were dried, filtered and concentrated. Purification with flash column chromatography (toluene/ethyl acetate 19:1) furnished **8c** (124 mg, 63%) as a colorless oil. ^1^H NMR (300 MHz, CDCl_3_): δ 1.88-1.98 (m, 1H), 2.23-2.32 (m, 1H), 2.73 (dd, *J* = 9.3, 13.8 Hz, 1H), 2.94-3.05 (m, 1H), 3.17 (dd, *J* = 4.5, 14.1 Hz, 1H), 3.45-3.57 (m, 2H), 3.79-3.84 (m, 1H), 4.43-4.53 (m, 3H), 4.55-4.66 (m, 2H), 7.14-7.17 (m, 2H), 7.23-7.37 (m, 13H); ^13^C NMR (75.5 MHz, CDCl_3_): δ 27.8, 37.1, 41.1, 69.0, 73.6, 73.7, 78.1, 78.5, 126.8, 127.8, 127.9, 128.0, 128.1, 128.6, 128.8, 129.1, 137.9, 138.5, 179.1.

#### (3S,5S)-5-((R)-1,2-Bis-benzyloxy-ethyl)-3-isopropyl-dihyd-ro-furan-2-one (8d)

Compound **8d **was synthesized in 41% yield from **7b** using 2-iodopropane instead of EtBr according to the synthesis method for compound **8b**. ^1^H NMR (300 MHz, CDCl_3_): δ 0.91 (d, *J* = 6.9 Hz, 3H), 0.99 (d, *J* = 6.9 Hz, 3H), 1.92-2.03 (m, 1H), 2.08-2.17 (m, 1H), 2.19-2.30 (m, 1H), 2.56-2.63 (m, 1H), 3.55-3.63 (m, 2H), 3.80-3.85 (m, 1H), 4.48-4.55 (m, 2H), 4.56-4.60 (m, 1H), 4.62-4.71 (m, 2H), 7.28-7.39 (m, 10H); ^13^C NMR (75.5 MHz, CDCl_3_): δ 18.4, 20.5, 24.2, 29.0, 45.5, 69.1, 73.6, 73.7, 78.0, 78.7, 127.8, 128.0, 128.1, 128.2, 128.6, 137.9, 138.0, 179.0. MS calcd (M+H)^+^: 369.2 found 368.7.

#### (3R,5S)-5-((R)-1,2-Dihydroxy-ethyl)-3-methoxy-dihydro-furan-2-one (9a)

To a solution of **8a** (979 mg, 2.75 mmol) in EtOH (95%, 10 mL) and AcOH (1 mL) Pd(OH)_2_ on active carbon (300 mg) was added. The mixture was hydrogenolyzed (atmospheric pressure) at room temperature overnight. Filtration through Celite, concentration and flash column chromatography (toluene/EtOAc 2:1) yielded **9a** (400 mg, 83%) as a colorless oil. [α]_D_^22^ +39 (*c* 0.1, MeOH); ^1^H NMR (300 MHz, CD_3_OD) δ 2.15 (ddd, *J *= 6.9, 8.2, 13.5 Hz, 1H), 2.59 (ddd, *J *= 3.9, 8.2, 13.5 Hz, 1H), 3.51 (s, 3H), 3.53-3.58 (m, 2H), 3.82 (app. dt, *J* = 3.6, 5.9 Hz, 1H), 4.22 (d, *J* = 6.9, 8.2 Hz, 1H), 4.68 (app. dt, *J* = 3.8, 8.2 Hz, 1H); ^13^C NMR (75.5 MHz, CD_3_OD) δ 30.1, 58.4, 63.6, 73.3, 77.0, 80.1, 177.3.

#### (3R,5S)-5-((R)-1,2-Dihydroxy-ethyl)-3-ethyl-dihydro-furan -2-one (9b)

A solution of **8b** (86 mg, 0.24 mmol) in EtOH (2 mL, 95%) was hydrogenolyzed using H_2_/Pd-C (1 atm.) for 4 h. The suspension was filtered through Celite and concentrated to give **9b** (41 mg, 98%) as a colorless oil. ^1^H NMR (300 MHz, CDCl_3_): δ 1.00 (t, *J* = 7.5 Hz, 3H), 1.44-1.57 (m, 1H), 1.77-1.89 (m, 1H), 1.91-2.02 (m, 1H), 2.42-2.51 (m, 1H), 2.59-2.70 (m, 1H), 3.60-3.66 (m, 1H), 3.72-3.77 (m, 1H), 3.82-3.89 (m, 1H), 4.45-4.51 (m, 1H); ^13^C NMR (75.5 MHz, CDCl_3_): δ 11.7, 24.4, 28.4, 40.8, 63.2, 72.6, 78.3, 180.1.

#### (3R,5S)-3-Benzyl-5-((R)-1,2-dihydroxy-ethyl)-dihydro-fur-an-2-one (9c)

Compound **9c **was synthesized from **8c** according to the synthesis method for compound **9b**. Purification using flash column chromatography (methanol/EtOAc 1:34) gave **9c** in 84% yield as a colorless oil. ^1^H NMR (300 MHz, CDCl_3_): δ 1.97-2.07 (m, 1H), 2.30-2.39 (m, 1H), 2.77 (dd, *J* = 9.0, 13.8 Hz, 1H), 2.98-3.08 (m, 1H), 3.18 (dd, *J* = 4.5, 13.8 Hz, 1H), 3.55-3.60 (m, 1H), 3.67-3.71 (m, 1H), 3.80-3.84 (m, 1H), 4.27-4.33 (m, 1H), 7.18-7.22 (m, 2H), 7.23-7.34 (m 3H); ^13^C NMR (75.5 MHz, CDCl_3_): δ 28.2, 37.0, 41.0, 61.2, 73.6, 77.9, 79.8, 126.9, 128.1, 128.3, 128.7, 128.8, 129.0, 137.6, 138.3, 178.9. MS calcd (M+H)^+^: 237.1; found 236.8.

#### (3S,5S)-5-((R>)-1,2-Dihydroxy-ethyl)-3-isopropyl-dihydro-furan-2-one (9d)

Compound **9d **was synthesized from **8d** according to the synthesis method for compound **9b**. Purification using flash column chromatography (EtOAc) gave **9d** (62%) as a colorless oil. ^1^H NMR (300 MHz, CDCl_3_): δ 0.94 (d, *J* = 6.9 Hz, 3H), 1.03 (d, *J* = 6.9 Hz, 3H), 2.04-2.18 (m, 2H), 2.29-2.39 (m, 1H), 2.60-2.68 (m, 1H), 3.64-3.68 (m, 1H), 3.75-3.84 (m, 2H), 4.40-4.46 (m, 1H); ^13^C NMR (75.5 MHz, CDCl_3_): δ 18.6, 20.5, 25.0, 29.1, 45.4, 63.2, 72.7, 78.2, 179.1.

#### (3R,5S)-5-((R)-1-Hydroxy-2-((4-methoxybenzyl)oxy)ethyl)-3-methoxy-dihydro-furan-2-one (10a)

Compound **9a** (163 mg, 0.93 mmol) and Bu_2_SnO (299 mg, 1.20 mmol) were added to toluene (6 mL) and the mixture was refluxed for 4 h. The temperature was lowered to 90 °C and tetrabutylammonium bromide (343 mg, 1.06 mmol) and 4-methoxybenzyl bromide (153 μL, 1.06 mmol) were added and the mixture was stirred overnight at 90 °C. Concentration and flash column chromatography (toluene/EtOAc 6:1) provided **10a** (246 mg, 90%) as a colorless oil. [α]_D_^22^ +18 (*c* 0.1, MeOH); ^1^H NMR (300 MHz, CDCl_3_) δ 2.17 (ddd, *J *= 6.0, 7.8, 13.7 Hz, 1H), 2.51 (ddd, *J *= 4.8, 8.0, 13.7 Hz, 1H), 2.63-2.68 (b, 1H), 3.43-3.57 (m, 2H), 3.53 (s, overlapped, 3H), 3.81 (s, 3H), 3.88-3.98 (m, 1H), 4.09 (dd, *J* = 6.0, 8.0 Hz, 1H), 4.45 (d, *J *= 11.5 Hz, 1H), 4.49 (d, *J *= 11.5 Hz, 1H), 4.54-4.62 (m, 1H), 6.88 (d, *J *= 8.8 Hz, 2H), 7.23 (d, *J *= 8.8 Hz, 2H); ^13^C NMR (75.5 MHz, CDCl_3_) δ 30.3, 55.4, 58.3, 70.1, 71.0, 73.4, 75.5, 78.1, 114.1, 129.6, 159.6, 174.7.

#### (3S,5S)-3-Ethyl-5-((R)-1-hydroxy-2-(4-methoxy-benzylox-y)-ethyl)-dihydro-furan-2-one (10b)

Compound **10b** (colorless oil) was synthesized in 70% yield from **9b** according to the synthesis method for compound **10a**. ^1^H NMR (300 MHz, CDCl_3_): δ 0.98 (t, *J* = 7.5 Hz, 3H), 1.42-1.57 (m, 1H), 1.77-1.87 (m, 1H), 1.88-1.98 (m, 1H), 2.39-2.48 (m, 1H), 2.54-2.61 (m, 1H), 3.50 (dd, *J* = 6.0, 9.9 Hz, 1H), 3.58 (dd, *J* = 4.2, 9.9 Hz, 1H), 3.80 (s, 3H), 3.83-3.86 (m, 1H), 4.40-4.45 (m, 1H), 4.47 (d, *J* = 2.7 Hz, 2H), 6.85-6.90 (m, 2H), 7.21-7.26 (m, 2H); ^13^C NMR (75.5 MHz, CDCl_3_) δ 11.7, 24.3, 28.6, 40.4, 55.4, 70.3, 71.2, 73.4, 77.8, 114.1, 129.6, 129.7, 159.6, 179.5.

#### (3R,5S)-3-Benzyl-5((R)-1-hydroxy-2-(4-methoxy-benzylo-xy)-ethyl)-dihydro-furan-2-one (10c)

Compound **10c** (colorless oil) was synthesized in 88% yield from **9c **according to the synthesis method for compound **10a**. ^1^H NMR (300 MHz, CDCl_3_): δ 1.93-2.03 (m, 1H), 2.27-2.36 (m, 1H), 2.75 (dd, *J* = 9.3, 13.8 Hz, 1H), 2.95-3.05 (m, 1H), 3.18 (dd, *J* = 4.5, 13.8 Hz, 1H), 3.42-4.54 (m, 2H), 3.80 (s, 3H), 3.81-3.86 (m, 1H), 4.28-4.34 (m, 1H), 4.42 (d, *J* = 11.4 Hz, 1H), 4.47 (d, *J* = 11.4 Hz, 1H), 6.85-6.90 (m, 2H), 7.18-7.35 (m, 7H); ^13^C NMR (75.5 MHz, CDCl_3_): δ 28.2, 36.8, 40.8, 55.4, 70.2, 71.1, 73.3, 78.0, 114.0, 126.9, 128.8, 128.9, 129.0, 129.6, 129.7, 138.3, 159.6, 178.9. MS calcd (M+Na)^+^: 379.2; found 379.6.

#### (3S,5S)-5-((R)-1-Hydroxy-2-(4-methoxy-benzyloxy)-ethyl)-3-isopropyl-dihydro-furan-2-one (10d)

Compound **10d** (colorless oil) was synthesized in 92% yield from **9d** according to the synthesis method for compound **10a**. ^1^H NMR (300 MHz, CDCl_3_): δ 0.90 (d, *J *= 6.6 Hz, 3H), 0.98 (d, *J *= 6.9 Hz, 3H), 1.94-2.04 (m, 1H), 2.05-2.16 (m, 1H), 2.22-2.32 (m, 1H), 2.54-2.62 (m, 1H), 3.45-3.56 (m, 2H), 3.77 (s, 3H), 3.81-3.88 (m, 1H), 4.36-4.42 (m, 1H), 4.45 (d, *J *= 3.6 Hz, 2H), 6.83-6.88 (m, 2H), 7.20-7.24 (m, 2H); ^13^C NMR (75.5 MHz, CDCl_3_): δ 18.5, 20.5, 24.9, 28.9, 45.1, 55.4, 70.3, 71.3, 73.4, 77.9, 114.1, 128.7, 129.6, 159.6, 178.9.

#### (3R,5S)-5-[(S)-1-Azido-2-(4-methoxy-benzyloxy)-ethyl]-3-methoxy-dihydro-furan-2-one (11a)>

Compound **10a** (53 mg, 0.18 mmol) and PPh_3_ (68 mg, 0.26 mmol) were dissolved in dry THF (2.5 mL). The mixture was cooled to –15 °C and DIAD (81 μL, 0.41 mmol) was added. After stirring for 10 min at –15 °C the temperature was raised to 0 °C and DPPA (60 μL, 0.28 mmol) was added. The solution was stirred for 30 min at 0 °C and then overnight at room temperature. Concentration and flash column chromatography (toluene/EtOAc 15:1) gave **11a** (57 mg, 99%) as a colorless oil. [α]_D_^22^ +92 (*c* 0.1, MeOH); ^1^H NMR (300 MHz, CDCl_3_) δ 2.24 (ddd, *J *= 5.8, 8.0, 13.7 Hz, 1H), 2.35-2.46 (m, 1H), 3.54 (s, 3H), 3.60-3.67 (m, 1H), 3.69-3.74 (m, 2H), 3.80 (s, 3H), 4.13 (dd, *J* = 5.8, 8.0 Hz, 1H), 4.50 (s, 2H), 4.66 (ddd, *J *= 3.0, 5.0, 8.0 Hz, 1H), 6.88 (d, *J *= 8.7 Hz, 2H), 7.25 (d, *J *= 8.7 Hz, 2H); ^13^C NMR (75.5 MHz, CDCl_3_) δ 32.4, 55.4, 58.4, 63.5, 69.4, 73.5, 74.9, 76.3, 114.1, 129.4, 129.6, 159.6, 174.0.

#### (3R,5S)-5-[(S)S)-1-Azido-2-(4-methoxy-benzyloxy)-ethyl]-3-ethyl-dihydro-furan-2-one (11b)>

Compound **11b** (colorless oil) was synthesized in 73% yield from **10b** according to the synthesis method for compound **11a**. ^1^H NMR (300 MHz, CDCl_3_) δ 0.99 (t, *J* = 7.5 Hz, 3H), 1.43-1.60 (m, 1H), 1.78-1.89 (m, 1H), 1.97-2.06 (m, 1H), 2.25-2.34 (m, 1H), 2.65-2.76 (m, 1H), 3.59-3.65 (m, 1H), 3.69-3.73 (m, 2H), 3.81 (s, 3H), 4.50 (s, 2H), 4.53-4.59 (m, 1H), 6.86-6.91 (m, 2H), 7.23-7.28 (m, 2H); ^13^C NMR (75.5 MHz, CDCl_3_): δ 11.6, 24.5, 30.3, 40.3, 55.4, 64.2, 69.5, 73.5, 76.3, 114.1, 129.5, 129.6, 159.6, 178.8.

#### (3R,5S)-5-[(S)S)-1-Azido-2-(4-methoxy-benzyloxy)-ethyl]-3-benzyl-dihydro-furan-2-one (11c)>

Compound **11c** (colorless oil) was synthesized in 74% yield from **10c** according to the synthesis method for compound **11a**. (The temperature was 0 °C instead of –15 °C.) ^1^H NMR (300 MHz, CDCl_3_): δ 2.02-2.22 (m, 2H), 2.72-2.79 (m, 1H), 3.06-3.14 (m, 1H), 3.16-3.22 (m, 1H), 3.54-3.59 (m, 1H), 3.68 (s, 1H), 3.70 (d, *J* = 2.4 Hz, 1H), 3.81 (s, 3H), 4.30-4.43 (m, 1H), 4.48 (s, 2H), 6.86-6.91 (m, 2H), 7.18-7.35 (m, 7H); ^13^C NMR (75.5 MHz, CDCl_3_): δ 29.9, 36.9, 40.6, 55.4, 64.0, 69.5, 73.5, 76.3, 114.0, 127.0, 128.9, 129.0, 129.5, 129.6, 138.1, 159.6, 178.2. 

#### (3S,5S)-5-[(S)S)-1-Azido-2-(4-methoxy-benzyloxy)-ethyl]-3-isopropyl-dihydro-furan-2-one (11d)

Compound **11d** (colorless oil) was synthesized in 57% yield from **10d** according to the synthesis method for compound **11c**. ^1^H NMR (300 MHz, CDCl_3_): δ 0.91 (d, *J* = 6.9 Hz, 3H), 1.00 (d, *J* = 6.9 Hz, 3H), 2.08-2.19 (m, 3H), 2.65-2.73 (m, 1H), 3.58-3.63 (m, 1H), 3.70-3.72 (m, 2H), 3.79 (s, 3H), 4.49 (s, overlapped, 2H), 4.48-4.53 (m, 1H), 6.87-6.90 (m, 2H), 7.23-7.28 (m, 2H); ^13^C NMR (75.5 MHz, CDCl_3_): δ 18.3, 20.3, 26.6, 29.0, 44.9, 55.3, 64.2, 69.4, 73.4, 76.3, 114.0, 129.5, 159.5, 178.1.

#### (3R,5S)-5-((S)-1-Azido-2-hydroxy-ethyl)-3-methoxy-dihydro-furan-2-one (12a)

To a solution of **11a** (321 mg, 1.00 mmol) in DCM (5 mL) and H_2_O (0.25 mL) DDQ (250 mg, 1.10 mmol) was added. The mixture was stirred overnight at room temperature and concentrated. Flash column chromatography (toluene/EtOAc 2:1) furnished **12a** (194 mg, 97%) as a colorless oil. [α]_D_^22^ +95 (*c* 0.1, MeOH); ^1^H NMR (300 MHz, CDCl_3_) δ 2.30 (ddd, *J *= 5.8, 7.7, 13.7 Hz, 1H), 2.43 (ddd, *J* = 4.9, 8.0, 13.7 Hz, 1H), 2.87 (bs, 1H), 3.53 (s, 3H), 3.60 (dt, *J* = 3.0, 6.3 Hz, 1H), 3.87 (d, *J *= 6.3 Hz, 1H), 4.16 (dd, *J *= 5.8, 8.0 Hz, 2H), 4.74 (ddd, *J *= 3.6, 5.5, 7.7 Hz, 1H); ^13^C NMR (75.5 MHz, CDCl_3_) δ 32.5, 58.6, 62.4, 65.5, 75.1, 76.8, 174.6.

#### (3R,5S)-5-((S)-1-Azido-2-hydroxy-ethyl)-3-ethyl-dihydro-furan-2-one (12b)

Compound **12b** (colorless oil) was synthesized in 94% yield from **11b** according to the synthesis method for compound **12a**. ^1^H NMR (300 MHz, CD_3_OD): δ 1.01 (t, *J* = 7.5 Hz, 3H), 1.45-1.60 (m, 1H), 1.74-1.87 (m, 1H), 2.09-2.18 (m, 1H), 2.32-2.41 (m, 1H), 2.67-2.77 (m, 1H), 3.57-3.63 (m, 1H), 3.71-3.83 (m, 2H), 4.61-4.67 (m, 1H); ^13^C NMR (75.5 MHz, CD_3_OD): δ 11.7, 25.4, 31.2, 41.7, 62.9, 67.8, 78.5, 181.3. MS calcd (M+H)^+^: 200.1; found 199.5.

#### (3R,5S)-5-((S)-1-Azido-2-hydroxy-ethyl)-3-benzyl-dihydro-furan-2-one (12c)>

Compound **12c** (colorless oil) was synthesized in 91% yield from **11c** according to the synthesis method for compound **12a**. ^1^H NMR (300 MHz, CDCl_3_): δ 2.12-2.20 (m, 2H), 2.75-2.82 (m, 1H), 3.07-3.14 (m, 1H), 3.17-3.22 (m, 1H), 3.49-3.54 (m, 1H), 3.84 (d, *J* = 5.7 Hz, 2H), 4.41-4.47 (m, 1H), 7.18-7.23 (m, 2H), 7.25-7.35 (m, 3H); ^13^C NMR (75.5 MHz, CDCl_3_): δ 30.0, 36.9, 40.7, 62.5, 65.9, 76.6, 127.1, 129.0, 129.1, 138.0, 178.2. MS calcd (M+Na)^+^: 284.1; found 283.6.

#### (3S,5S)-5-((S)-1-Azido-2-hydroxy-ethyl)-3-isopropyl-dihydro-furan-2-one (12d)

Compound **12d** (yellow powder) was synthesized in quantitative yield from **11d** according to the synthesis method for compound **12a**. ^1^H NMR (300 MHz, CD_3_OD): δ 0.94 (d, *J* = 6.9 Hz, 3H), 1.03 (d, *J* = 6.6 Hz, 3H), 2.04-2.15 (m, 1H), 2.21-2.26 (m, 2H), 2.70-2.77 (m, 1H), 3.56-3.62 (m, 1H), 3.71-3.83 (m, 2H), 4.57-4.63 (m, 1H); ^13^C NMR (75.5 MHz, CD_3_OD): δ 18.7, 20.4, 27.7, 30.3, 46.4, 62.9, 67.9, 78.5, 180.6.

### General Procedures for the Synthesis of Target Compounds

#### (3R,5S)-5-{(S)-1-Azido-2-[4'-fluoro-3'-(3-methoxy-propoxy)-biphenyl-4-yloxy]-ethyl}-3-methoxy-dihydro-furan-2-one (13)

##### General Procedure A. Debenzylation

To a solution of **3** (121 mg, 0.330 mmol) in EtOH (95%, 4 mL) was added Pd-C (10%, 60 mg) and the mixture was hydrogenolyzed (atmospheric pressure) at room temperature for 3h. The suspension was filtered through Celite and concentrated. The resulting phenol was used without any further purification in the following step.

##### General Procedure B. Substitution

To a cooled (0 °C) solution of **12a** (60 mg, 0.298 mmol) and dry pyridine (46 μL, 0.571 mmol) in dry DCM (3 mL) was added Tf_2_O (84 μL, 0.498 mmol) and the mixture was stirred at 0 °C for 45 min. The reaction was quenched with saturated aqueous NH_4_Cl and the organic phase was separated, dried, filtered and concentrated. 

The phenol and the triflate (obtained from the general procedures A and B, respectively) were dissolved in dry DCM (3 mL) and stirred together with 4 Å powdered molecular sieves (50 mg) for 10 min before the addition of Cs_2_CO_3_ (215 mg, 0.66 mmol). The mixture was heated to 40 °C and stirred for 1.5 h under N_2_ atmosphere, filtered, and concentrated. Purification using LC-MS gave colorless semicrystalline **13** (64 mg, 47%). [α]_D_^22^ +115 (*c* 0.1, MeOH); ^1^H NMR (300 MHz, CDCl_3_) δ 1.92-2.02 (m, 2H), 2.30-2.41 (m, 1H), 2.48-2.59 (m, 1H), 3.30 (s, 3H), 3.44 (t, *J *= 6.3 Hz, 2H), 3.58 (s, 3H), 3.92 (dt, *J *= 2.8, 6.2 Hz, 1H), 4.03 (t, *J *= 6.3 Hz, 2H), 4.19 (dd, *J* = 6.1, 8.0 Hz, 1H), 4.29 (d, *J *= 6.3 Hz, 2H), 4.77-4.84 (m, 1H), 6.53-6.60 (m, 2H), 7.05 (app. t, *J*_HH_= *J*_HF_ = 8.8 Hz, 2H), 7.20 (d, *J *= 8.5 Hz, 1H), 7.43 (dd, *J*_HF_**= 5.5, *J*_HH_ 8.8 Hz, 2H); ^13^C NMR (75.5 MHz, CDCl_3_) δ 29.5, 32.5, 58.5, 58.8, 63.0, 65.5, 68.1, 69.2, 74.9, 76.1, 100.6, 105.9, 114.8 (d, *J*_CF_ = 21.2 Hz, 2C), 124.0, 131.1 (d, *J*_CF_ = 8.0 Hz, 2C), 131.3, 134.2, 157.0, 158.5, 161.9 (d, *J*_CF_ = 245.1 Hz), 173.8. MS calcd (M+H)^+^: 460.2; found 460.1.

#### (2R,4S,5S)-5-Azido-6-[4'-fluoro-2-(3-methoxy-propoxy)-biphenyl-4-yloxy]-4-hydroxy-2-methoxy-hexanoic acid [(1S,2S)-1-(4-chloro-benzylcarbamoyl)-2-methyl-butyl]-amide (14)

##### General Procedure C. Lactone Opening

2-Hydroxypyridine (20 mg, 0.210 mmol) was added to a solution of **13** (28 mg, 0.061 mmol) and amine **O** (40 mg, 0.157 mmol) in DIPEA (4 mL) and DMF (0.5 mL). The mixture was stirred at 75 °C under N_2_ atmosphere overnight. Concentration and purification using LC-MS gave **14** (36 mg, 83%) as a colorless solid. [α]_D_^22^ +15 (*c* 0.1, MeOH); ^1^H NMR (300 MHz, CDCl_3_) δ 0.88-1.00 (m, 6H), 1.03-1.19 (m, 1H), 1.41-1.56 (m, 1H), 1.91-2.03 (m, 3H), 2.07-2.23 (m, 2H), 2.90 (bs, 1H), 3.29 (s, 3H), 3.43 (t, overlapped, *J* = 6.1 Hz, 2H), 3.46 (s, 3H), 3.64-3.72 (m, 1H), 3.87 (dd, *J *= 4.1, 5.5 Hz, 1H), 4.02 (t, *J *= 6.2 Hz, 2H), 4.06-4.22 (m, 3H), 4.29-4.38 (m, 3H), 6.52 (dd, *J *= 2.5, 8.2 Hz, 1H), 6.57 (d, *J *= 2.2 Hz, 1H), 7.00-7.10 (m, 3H), 7.14-7.28 (m, 6H), 7.44 (dd, *J*_HF_**= 5.5, *J*_HH_ 8.8 Hz, 2H); ^13^C NMR (75.5 MHz, CDCl_3_) δ 11.6, 16.2, 24.8, 29.5, 35.6, 36.4, 42.9, 57.8, 58.5, 58.8, 65.0, 65.5, 67.4, 68.3, 69.2, 79.5, 100.8, 105.7, 114.8 (d, *J*_CF_ = 21.2 Hz, 2C), 123.7, 128.9, 129.3, 131.1 (d, *J*_CF_ = 8.0 Hz, 2C), 131.2, 133.4, 134.3, 137.0, 156.9, 158.9, 161.9 (d, *J*_CF_ = 245.4 Hz), 171.1, 172.5. MS calcd (M+H)^+^: 714.3; found 714.2. 

#### (2R,4S,5S)-5-Amino-6-[4'-fluoro-2-(3-methoxy-propoxy)-biphenyl-4-yloxy]-4-hydroxy-2-methoxy-hexanoic acid [(1-S,2S)-1-(4-chloro-benzylcarbamoyl)-2-methyl-butyl]-amide (15)

##### General Procedure D. Azide Reduction

To a solution of **14** (31 mg, 0.043 mmol) in MeOH (4 mL), THF (1 mL), and a few drops of water was added PPh_3_ (28 mg, 0.107 mmol) and the mixture was stirred at room temperature overnight. Concentration and flash column chromatography (EtOAc/MeOH 9:1 + 1% TEA) gave **15** (25 mg, 84%) as a colorless solid. [α]_D_^22^ +4.0 (*c* 0.1, MeOH); ^1^H NMR (300 MHz, CDCl_3_) δ 0.88-1.00 (m, 6H), 1.04-1.19 (m, 1H), 1.41-1.55 (m, 1H), 1.90-2.01 (m, 3H), 2.06-2.19 m, 1H), 2.20-2.31 m, 1H), 2.47 (bs, 2H), 2.99-3.08 (m, 1H), 3.29 (s, 3H), 3.43 (t, overlapped, *J *= 6.0 Hz, 2H), 3.45 (s, 3H), 3.77-3.92 (m, 3H), 3.94-4.00 (m, 1H), 4.02 (t, overlapped, *J *= 6.3 Hz, 2H), 4.32-4.37 (m, 2H), 4.38-4.45 (m, 1H), 6.50 (dd, *J *= 2.3, 8.4 Hz, 1H), 6.55 (d, *J *= 2.3 Hz, 1H), 6.97 (d, *J *= 9.1 Hz, 1H), 7.05 (app. t, *J*_HH_**= *J*_HF_ = 8.8 Hz, 2H), 7.14-7.28 (m, 5H), 7.43 (dd, *J*_HF_**= 5.5, *J*_HH_ = 9.1 Hz, 2H), 7.57-7.66 (b, 1H); ^13^C NMR (75.5 MHz, CDCl_3_) δ 11.9, 16.3, 24.7, 29.6, 35.8, 36.0, 42.9, 54.7, 57.8, 58.4, 58.8, 65.5, 66.6, 69.2, 70.1, 79.6, 100.6, 105.8, 114.8 (d, *J*_CF_ = 21.2 Hz, 2C), 123.4, 128.8, 129.4, 131.0 (d, *J*_CF_ = 8.0 Hz, 2C), 131.2, 133.1, 134.3, 137.2, 156.9, 159.3, 161.8 (d, *J*_CF_ = 245.1 Hz), 171.1, 172.8. HRMS calcd (M+H)^+^: 688.3165; found 688.3197. LC-MS Purity System A: *t*_R_ = 6.72 min, 100%; System B: *t*_R_ = 5.71 min, 98%.

#### N-((2S,3S,5R)-6-((2S,3S)-1-(4-chlorobenzylamino)-3-methyl-1-oxopentan-2-ylamino)-1-(4'-fluoro-2-(3-methoxypropoxy)biphenyl-4-yloxy)-3-hydroxy-5-methoxy-6-oxohexan-2-yl)-3-(N-methylmethylsulfonamido)benzamide(16)

##### General Procedure E. Peptide Coupling

To a cooled (0 °C) solution of acid **A** (5.0 mg, 0.022 mmol), amine **15** (17 mg, 0.025 mmol) and DIPEA (12 μL, 0.069 mmol) in DMF (1 mL) was added HATU (10 mg, 0.026 mmol). The solution was stirred for 0.5 h at 0 °C and for an additional 2 h at room temperature. EtOAc and brine were added and the organic phase was separated and washed once with brine, dried and concentrated. Purification using LC-MS gave **16** (12 mg, 61%) as a colorless solid. [α]_D_^22^ -7.0 (*c* 0.1, MeOH); ^1^H NMR (300 MHz, CDCl_3_) δ 0.88-0.98 (m, 6H), 1.04-1.20 (m, 1H), 1.41-1.56 (m, 1H), 1.81 (bs, 1H), 1.90-2.01 (m, 2H), 2.02-2.11 (m, 2H), 2.12-2.24 (m, 1H), 2.85 (s, 3H), 3.28 (s, 3H), 3.35 (s, 3H), 3.43 (t, overlapped, *J *= 6.2 Hz, 2H), 3.46 (s, 3H), 3.86-3.92 (m, 1H), 4.02 (t, *J *= 6.2 Hz, 2H), 4.12-4.21 (m, 2H), 4.25-4.43 (m, 5H), 6.56-6.62 (m, 2H), 6.90-6.99 (m, 2H), 7.01-7.10 (m, 3H), 7.12-7.23 (m, 5H), 7.39-7.50 (m, 3H), 7.53-7.58 (b, 1H), 7.67-7.72 (b, 1H), 7.86 (s, 1H), ^13^C NMR (75.5 MHz, CDCl_3_) δ 11.6, 16.1, 24.9, 29.6, 35.7, 35.8, 36.6, 38.2, 43.0, 53.7, 57.9, 58.5, 58.8, 65.5, 66.7, 67.3, 69.3, 79.8, 100.1, 106.1, 114.8 (d, *J*_CF_ = 21.2 Hz, 2C), 123.5, 125.0, 125.9, 128.9, 129.3, 129.8, 131.1 (d, *J*_CF_ = 7.7 Hz, 2C), 131.3, 133.4, 134.6, 135.5, 136.8, 142.1, 157.0, 159.1, 161.9 (d, *J*_CF_ = 245.1 Hz), 167.1, 171.2, 172.8. HRMS calcd (M+H)^+^: 899.3468; found 899.3486. LC-MS Purity System A: *t*_R_ = 10.49 min, 98%; System B: *t*_R_ = 6.61 min, 97%.

#### (2R,4S,5S)-5-Amino-6-(3-benzyloxy-phenoxy)-4-hydroxy-2-methoxy-hexanoic acid ((S)-1-benzylcarbamoyl-2-methyl-propyl)-amide (17)

Compound **17** (colorless solid) was synthesized from **12a** according to the general procedures B-D using phenol **C** and amine **M**. [α]_D_^22^ -2.0 (*c* 0.1, MeOH); ^1^H NMR (300 MHz, CD_3_OD) δ 0.93 (d, *J *= 6.9 Hz, 3H), 0.95 (d, *J *= 6.9 Hz, 3H), 1.90-2.02 (m, 2H), 2.14-2.26 (m, 1H), 2.91-3.00 (m, 1H), 3.38 (s, 3H), 3.79-3.99 (m, 4H), 4.24 (d, *J *= 6.6 Hz, 1H), 4.31 (d, *J *= 14.8 Hz, 1H), 4.37 (d, *J *= 14.8 Hz, 1H), 5.01 (s, 2H), 6.48-6.60 (m, 3H), 7.10-7.43 (m, 11H); ^13^C NMR (75.5 MHz, CD_3_OD) δ 18.2, 19.8, 31.3, 36.9, 43.9, 55.0, 58.5, 59.1, 68.3, 70.3, 70.7, 80.3, 102.8, 107.9, 108.1, 127.9, 128.1, 128.3, 128.5, 129.1, 130.6, 137.9, 139.0, 160.7, 160.9, 172.6, 174.4. HRMS calcd (M+H)^++^: 564.3074; found 564.3074. LC-MS Purity System A: *t*_R_ = 4.18 min, 100%; System B: *t*_R_ = 2.29 min, 98%.

#### N-[(1S,2S,4R)-4-((S)-1-Benzylcarbamoyl-2-methyl-propy-lcarbamoyl)-1-(3-benzyloxy-phenoxymethyl)-2-hydroxy-4-methoxy-butyl]-3-(methanesulfonyl-methyl-amino)-benza-mide (18)

Compound **18 **(colorless solid) was synthesized from **17** according to the general procedure E. [α]_D_^22^ -10 (*c* 0.1, MeOH); ^1^H NMR (300 MHz, CD_3_OD) δ 0.86-0.96 (m, 6H), 1.91-2.01 (m, 2H), 2.03-2.17 (m, 1H), 2.89 (s, 3H), 3.31 (s, 3H), 3.36 (s, 3H), 3.92-3.99 (m, 1H), 4.09-4.29 (m, 4H), 4.34 (s, 2H), 4.51 (dt, *J *= 2.5, 6.6 Hz, 1H), 5.02 (s, 2H), 6.53-6.63 (s, 3H), 7.11-7.22 (m, 2H), 7.23-7.43 (m, 9H), 7.48 (t, *J *= 8.0 Hz, 1H), 7.60 (d, *J *= 8.0 Hz, 1H), 7.78 (d, *J *= 8.0 Hz, 1H), 7.90 (s, 1H); ^13^C NMR (75.5 MHz, CD_3_OD) δ 18.6, 19.8, 31.9, 35.7, 38.0, 38.5, 44.1, 54.2, 58.6, 59.9, 67.7, 68.0, 71.0, 80.5, 103.2, 108.4, 108.7, 126.5, 127.3, 128.3, 128.6, 128.6, 128.8, 129.5, 129.5, 130.5, 131.0, 131.1, 136.8, 138.7, 139.7, 143.5, 161.3, 161.5, 169.8, 173.3, 175.0. HRMS calcd (M+H)^+^: 775.3377; found 775.3391. LC-MS Purity System A: *t*_R_ = 9.77 min, 95%; System B: *t*_R_ = 5.74 min, 99%.

#### N-[(1S,2S,4R)-4-((S)-1-Benzylcarbamoyl-2-methyl-propylcarbamoyl)-1-(4-benzyloxy-phenoxymethyl)-2-hydroxy-4-me thoxy-butyl]-3-(methanesulfonyl-methyl-amino)-benzamide (19)

Compound **19** (colorless solid) was synthesized from **12a** according to the general procedures B-E using phenol **D** and amine **M**. [α]_D_^22^ +4.0 (*c* 0.1, MeOH); ^1^H NMR (300 MHz, CD_3_OD) δ 0.88 (d, *J *= 6.9 Hz, 3H), 0.91 (d, *J *= 6.9 Hz, 3H), 1.91-1.99 (m, 2H), 2.03-2.17 (m, 1H), 2.90 (s, 3H), 3.32 (s, 3H), 3.36 (s, 3H), 3.90-3.98 (m, 1H), 4.06-4.28 (m, 4H), 4.34 (s, 2H), 4.48 (dt, *J *= 2.5, 6.6 Hz, 1H), 5.00 (s, 2H), 6.89 (app. s, 3H), 7.09-7.43 (m, 11H), 7.49 (t, *J *= 7.8 Hz, 1H), 7.61 (d, *J *= 8.0 Hz, 1H), 7.78 (d, *J *= 8.0 Hz, 1H), 7.89 (s, 1H); ^13^C NMR (75.5 MHz, CD_3_OD) δ 18.6, 19.8, 31.9, 35.7, 38.1, 38.5, 44.1, 54.2, 58.6, 59.9, 67.7, 68.7, 71.6, 80.5, 116.9, 117.0, 126.5, 127.3, 128.3, 128.5, 128.6, 128.8, 129.4, 129.5, 130.4, 131.1, 136.8, 139.0, 139.7, 143.5, 154.4, 154.7, 169.8, 173.3, 175.0. HRMS calcd (M+H)^+^: 775.3377; found 775.3390. LC-MS Purity System A: *t*_R_ = 9.58 min, 96%; System B: *t*_R_ = 5.66 min, 97%.

#### (2R,4S,5S)-5-Amino-6-[4'-fluoro-2-(3-methoxy-propoxy)-bi phenyl-4-yloxy]-4-hydroxy-2-methoxy-hexanoic acid [(S)-1-(4-chloro-benzylcarbamoyl)-2-methyl-propyl]-amide (20)

Compound **20** (colorless solid) was synthesized from **12a** according to the general procedures A-D using amine **N**. [α]_D_^22^ +2.0 (*c* 0.1, MeOH); ^1^H NMR (300 MHz, CDCl_3_) δ 0.92 (d, *J *= 6.9 Hz, 3H), 0.99 (d, *J *= 7.1 Hz, 3H), 1.89-2.02 (m, 4H), 2.03-2.19 (m, 2H), 2.49-2.63 (m, 1H), 2.96-3.03 (m, 1H), 3.29 (s, 3H), 3.43 (t, overlapped, *J *= 6.2 Hz, 2H), 3.46 (s, 3H), 3.75-3.84 (m, 2H), 3.86-3.98 (m, 2H), 4.02 (t, *J *= 6.3 Hz, 2H), 4.32-4.41 (m, 3H), 6.50 (dd, *J *= 2.3, 8.4 Hz, 1H), 6.55 (d, *J *= 2.3 Hz, 1H), 6.94 (d, *J *= 8.8 Hz, 1H), 7.05 (app. t, *J*_HH_**= *J*_HF_ = 8.8 Hz, 2H), 7.15-7.29 (m, 5H), 7.44 (dd, *J*_HF_**= 5.5, *J*_HH_ 8.5 Hz, 2H), 7.54-7.62 (b, 1H); ^13^C NMR (75.5 MHz, CDCl_3_) δ 17.3, 19.8, 29.4, 29.6, 35.8, 42.9, 54.7, 58.0, 58.5, 58.8, 65.5, 66.6, 69.2, 70.1, 79.6, 100.7, 105.8, 114.8 (d, *J*_CF_ = 21.2 Hz, 2C), 123.5, 128.8, 129.4, 131.1 (d, *J*_CF_ = 8.0 Hz, 2C), 131.3, 133.2, 134.3, 137.2, 156.9, 159.3, 161.9 (d, *J*_CF_ = 245.4 Hz), 171.1, 172.9. HRMS calcd (M+H)^+^: 674.3008; found 674.3030. LC-MS Purity System A: *t*_R_ = 6.31 min, 100%; System B: *t*_R_ = 5.25 min, 98%.

#### (2R,4S,5S)-5-Amino-4-hydroxy-2-methoxy-6-[2-(3-methox-y-propoxy)-biphenyl-4-yloxy]-hexanoic acid [(1S,2S)-1-(4-chloro-benzylcarbamoyl)-2-methyl-butyl]-amide (21)

Compound **21** (colorless solid) was synthesized from **12a** according to the general procedures A-D using compound **4**. [α]_D_^22^ +12 (*c* 0.1, MeOH); ^1^H NMR (300 MHz, CDCl_3_) δ 0.91-0.99 (m, 6H), 1.02-1.20 (m, 1H), 1.40-1.55 (m, 1H), 1.89-2.02 (4H), 2.03-2.18 (m, 2H), 2.22-2.37 (m, 1H), 2.94-3.03 (m, 1H), 3.29 (s, 3H), 3.45 (t, overlapped, *J *= 6.2 Hz, 2H), 3.46 (s, 3H), 3.75-3.83 (m, 2H), 3.86-3.91 (m, 1H), 3.92-3.98 (m, 1H), 4.02 (t, *J *= 6.2 Hz, 2H), 4.33-4.37 (m, 1H), 4.38-4.44 (m, 2H), 6.51 (dd, *J *= 2.3, 8.4 Hz, 1H), 6.56 (d, *J *= 2.3 Hz, 1H), 6.92 (d, *J *= 8.8 Hz, 1H), 7.15-7.31 (m, 6H), 7.32-7.40 (m, 2H), 7.45-7.51 (m, 2H), 7.52-7.61 (b, 1H); ^13^C NMR (75.5 MHz, CDCl_3_) δ 12.0, 16.4, 24.7, 30.0, 35.7, 36.0, 43.0, 54.7, 57.9, 58.5, 58.8, 65.5, 66.5, 69.3, 70.1, 79.6, 100.7, 105.8, 124.6, 126.6, 128.0, 128.8, 129.4, 129.6, 131.4, 133.2, 137.2, 138.4, 157.0, 159.2, 171.1, 172.8. HRMS calcd (M+H)^+^: 670.3259; found 670.3284. LC-MS Purity System A: *t*_R_ = 6.55 min, 98%; System B:* t*_R_ = 5.62 min, 100%.

#### (2R,4S,5S)-5-Amino-4-hydroxy-2-methoxy-6-[3-(3-methox-y-propoxy)-phenoxy]-hexanoic acid [(1S,2S)-1-(4-chloro-benzylcarbamoyl)-2-methyl-butyl]-amide (22)

Compound **22** (colorless solid) was synthesized from **12a** according to the general procedures A-D using 1-benzyloxy-3-(3-methoxy-propoxy)-benzene****(benzylated** G**).****[α]_D_^22^ +9.0 (*c* 0.1, MeOH); ^1^H NMR (300 MHz, CDCl_3_) δ 0.88-0.98 (m, 6H), 1.01-1.18 (m, 1H), 1.39-1.54 (m, 1H), 1.88-2.16 (m, 4H), 2.17-2.38 (m, 4H), 2.95-3.02 (m, 1H), 3,47 (s, 3H), 3.43 (s, 3H), 3.55 (t, *J *= 6.2 Hz, 2H), 3.72-3.83 (m, 2H), 3.83-3.95 (m, 2H), 4.03 (t, *J *= 6.3 Hz, 2H), 4.34 (dd, *J *= 2.5, 5.8 Hz, 2H), 4.40 (dd, *J *= 4.5, 8.9 Hz, 1H), 6.43-6.50 (m, 2H), 6.53 (dd, *J *= 2.2, 8.2 Hz, 1H), 6.94 (d, *J *= 8.8 Hz, 1H), 7.12-7.29 (m, 5H), 7.61-7.69 (b, 1H); ^13^C NMR (75.5 MHz, CDCl_3_) δ 11.9, 16.3, 24.7, 29.7, 35.6, 36.1, 42.9, 54.7, 57.9, 58.4, 58.8, 65.1, 66.4, 69.4, 69.7, 79.4, 101.9, 106.9, 107.4, 128.8, 129.4, 130.1, 133.1, 137.2, 159.8, 160.5, 171.1, 172.9. HRMS calcd (M+H)^+^: 594.2946; found 594.2960. LC-MS Purity System A: *t*_R_ = 5.78 min, 100%; System B: *t*_R_ = 3.95 min, 98%.

#### (2R,4S,5S)-5-Amino-6-(4'-fluoro-biphenyl-4-yloxy)-4-hydroxy-2-methoxy-hexanoic acid [(1S,2S)-1-(4-chloro-benzylcarbamoyl)-2-methyl-butyl]-amide (23)

Compound **23** (colorless solid) was synthesized from **12a** according to the general procedures B-D using phenol **H**. [α]_D_^22^ +7.0 (*c* 0.1, MeOH); ^1^H NMR (300 MHz, CDCl_3_) δ 0.89-0.99 (m, 6H), 1.01-1.18 (m, 1H), 1.40-1.54 (m, 1H), 1.91-2.03 (m, 1H), 2.04-2.28 (m, 2H), 2.44 (s, 3H), 3.06-3.14 (m, 1H), 3.44 (s, 3H), 3.81-3.92 (m, 3H), 4.00 (dd, *J *= 4.8, 9.5 Hz, 2H), 4.35 (d, *J *= 5.8 Hz, 1H), 4.40 (dd, *J *= 4.7, 9.1 Hz, 1H), 6.92 (d, *J *= 9.1 Hz, 2H), 7.00 (d, *J *= 9.1 Hz, 1H), 7.09 (app. t, *J*_HH_**= *J*_HF_ = 8.7 Hz, 2H), 7.14-7.29 (m, 4H), 7.41-7.51 (m, 4H), 7.60-7.67 (b, 1H), ^13^C NMR (75.5 MHz, CDCl_3_) δ 11.9, 16.3, 24.7, 35.6, 36.2, 42.9, 54.8, 57.9, 58.4, 66.3, 69.5, 79.4, 115.1, 115.7 (d, *J*_CF_ = 21.5 Hz, 2C), 128.3, 128.4 (d, *J*_CF_ = 8.0 Hz, 2C), 128.8, 129.4, 133.2, 133.7, 136.9, 137.5, 158.0, 162.3 (d, *J*_CF_ = 245.9 Hz), 171.2, 172.9. HRMS calcd (M+H)^+^: 600.2641; found 600.2653. LC-MS Purity System A: *t*_R_ = 6.10 min, 98%; System B: *t*_R_ = 5.37 min, 96%.

#### N-{(1S,2S,4R)-4-[(1S,2S)-1-(4-Chloro-benzylcarbamoyl)-2-methyl-butylcarbamoyl]-2-hydroxy-4-methoxy-1-[2-(3-methoxy-propoxy)-biphenyl-4-yloxymethyl]-butyl}-3-(methan-esulfonyl-methyl-amino)-benzamide (24)

Compound **24 **(colorless solid) was synthesized from **21** according to the general procedure E. [α]_D_^22^ -2.0 (*c* 0.1, MeOH); ^1^H NMR (300 MHz, CDCl_3_) δ 0.85-0.98 (m, 6H), 1.03-1.20 (m, 1H), 1.39-1.55 (m, 1H), 1.74-2.01 (m, 3H), 2.02-2.24 (m, 3H), 2.85 (s, 3H), 3.29 (s, 3H), 3.35 (s, 3H), ), 3.44 (t, overlapped, *J *= 6.3 Hz, 2H), 3.46 (s, 3H), 3.85-3.91 (m, 1H), 4.02 (t, *J *= 6.3 Hz, 2H), 4.14-4.20 (m, 2H), 4.25-4.44 (m, 5H), 6.57-6.62 (m, 2H), 6.93 (d, *J *= 8.0 Hz, 1H), 6.99-7.10 (m, 2H), 7.11-7.23 (m, 4H), 7.24-7.32 (m, 2H), 7.33-7.41 (m, 2H), 7.45-7.52 (m, 3H), 7.57 (d, *J *= 7.1 Hz, 1H), 7.67 (d, *J *= 8.0 Hz, 1H), 7.87 s, 1H); ^13^C NMR (75.5 MHz, CDCl_3_) δ 11.6, 16.1, 25.0, 29.6, 35.7, 35.9, 36.6, 38.1, 43.0, 53.7, 57.9, 58.5, 58.8, 65.5, 66.9, 67.4, 69.3, 79.9, 100.7, 106.1, 124.6, 124.9, 125.7, 126.6, 128.0, 128.9, 129.3, 129.6, 129.7, 130.0, 131.5, 133.4, 135.5, 136.8, 138.4, 142.2, 157.0, 159.0, 167.0, 171.1, 172.8. HRMS calcd (M+H)^+^: 881.3562; found 881.3574. LC-MS Purity System A: *t*_R_ = 10.68 min, 98%; System B: *t*_R_ = 6.58 min, 97%.

#### N-{(1S,2S,4R)-4-[(1S,2S)-1-(4-Chloro-benzylcarbamoyl)-2-methyl-butylcarbamoyl]-2-hydroxy-4-methoxy-1-[2-(3-methoxypropoxy)-biphenyl-4-yloxymethyl]-butyl}-5-(methanesulfonyl-methyl-amino)-N'-((R)-1-phenyl-ethyl)-isophthalamide (25)

Compound **25** (colorless solid) was synthesized from **21** according to the general procedure E using acid **B**. [α]_D_^22^ -14 (*c* 0.1, MeOH); ^1^H NMR (300 MHz, CDCl_3_) δ 0.86-0.98 (m, 6H), 1.01-1.18 (m, 1H), 1.39-1.53 (m, 1H), 1.52 (d, *J *= 7.1 Hz, 3H), 1.89-2.09 (m, 4H), 2.11-2.24 (m, 1H), 2.84 (s, 3H), 3.27 (s, 3H), 3.32 (s, 3H), 3.43 (t, overlapped, *J *= 6.3 Hz, 2H), 3.45 (s, 3H), 3.68 (bs, 1H), 3.79-3.85 (m, 1H), 4.00 (t, *J *= 6.2 Hz, 2H), 4.08-4.23 (m, 3H), 4.26-4.41 (m, 4H), 5.20-5.32 (m, 1H), 6.57-6.62 (m, 2H), 7.03-7.14 (m, 5H), 7.15-7.43 (m, 12H), 7.47 (d, *J *= 7.7 Hz, 2H), 7.95 (s, 1H), 7.98 (s, 1H), 8.19 (s, 1H); ^13^C NMR (75.5 MHz, CDCl_3_) δ 11.6, 16.1, 22.0, 25.0, 29.6, 35.3, 35.8, 36.7, 38.1, 42.9, 50.1, 54.0, 57.8, 58.5, 58.8, 65.6, 66.6, 67.1, 69.4, 79.5, 100.8, 106.1, 124.6, 126.5, 126.6, 127.7, 128.0, 128.8, 128.9, 129.5, 129.7, 131.5, 133.2, 135.8, 136.0, 137.0, 138.5, 142.4, 143.2, 157.1, 159.1, 165.0, 166.5, 171.2, 173.1. HRMS calcd (M+H)^+^: 1028.4246; found 1028.4200. LC-MS Purity System A: *t*_R_ = 11.42 min, 100%; System B: *t*_R_ = 7.13 min, 100%.

#### (2R,4S,5S)-5-amino-N-((2S,3S)-1-(4-chlorobenzylamino)-3-methyl-1-oxopentan-2-yl)-2-ethyl-6-(4'-fluoro-2-(3-methoxypropoxy)biphenyl-4-yloxy)-4-hydroxyhexanamide (26)

Compound **26** (white solid) was synthesized from **12b **according to the general procedures A-D. [α]_D_^22^ -30 (*c* 0.1, MeOH); ^1^H NMR (300 MHz, CD_3_OD): δ 0.84-0.96 (m, 9H), 1.10-1.28 (m, 2H), 1.44-1.67 (m, 4H), 1.79-1.96 (m, 3H), 2.50-2.60 (m, 1H), 2.98 (q, *J* = 5.8 Hz, 1H), 3.27 (s, 3H), 3.43 (t, *J *= 6.3 Hz, 2H), 3.62-3.68 (m, 1H), 3.90-3.95 (m, 1H), 3.99-4.08 (m, 3H), 4.24 (d, *J *= 8.8 Hz, 1H), 4.34 (d, *J *= 7.7 Hz, 2H), 6.58-6.65 (m, 2H), 7.06 (t, *J* = 8.9 Hz, 2H), 7.17 (d, *J* = 8.2 Hz, 1H), 7.24-7.41 (m, 4H), 7.42-7.7.46 (m, 2H); ^13^C NMR (75.5 MHz, CD_3_OD): δ 11.1, 12.3, 16.0, 26.2, 27.8, 30.4, 37.6, 37.8, 43.3, 46.3, 56.3, 58.9, 59.3, 66.4, 70.2, 70.3, 70.6, 101.7, 107.3, 115.4 (d, *J*_CF_ = 21.2 Hz, 2C), 124.3, 129.5, 130.3, 132.0, 132.2 (d, *J*_CF_ = 7.7 Hz, 2C), 134.0, 136.0, 136.1, 138.7, 158.1, 160.9, 163.1 (d, *J*_CF_ = 244.6 Hz, 1C), 173.8, 178.3. HRMS calcd (M+H)^+^: 686.3372; found 686.3376. LC-MS Purity System A: *t*_R_ = 6.92 min, 100%; System B: *t*_R_ = 5.58 min, 100%.

#### (2R,4S,5S)-5-amino-2-benzyl-N-((2S,3S)-1-(4-chlorobenzy-lamino)-3-methyl-1-oxopentan-2-yl)-6-(4'-fluoro-2-(3-methoxypropoxy)biphenyl-4-yloxy)-4-hydroxyhexanamide (27)

Compound **27** (white solid) was synthesized from **12c** according to the general procedures A-D. [α]_D_^22^ -5 (*c* 0.1, MeOH); ^1^H NMR (300 MHz, CD_3_OD): δ 0.83-0.88 (m, 6H), 1.12-1.26 (m, 1H), 1.46-1.54 (m, 1H), 1.64-1.72 (m, 1H), 1.79-1.86 (m, 1H), 1.92 (t, *J* = 6.3 Hz, 2H), 2.70-2.76 (m, 1H), 2.88-3.00 (m, 3H), 3.27 (s, 3H), 3.43 (t, *J* = 6.3 Hz, 2H), 3.65-3.71 (m, 1H), 3.88-3.93 (m, 1H), 4.02 (t, overlapped, *J* = 6.3 Hz, 2H), 4.03-4.05 (m, 1H), 4.19 (d, *J* = 8.1 Hz, 1H), 4.26 (s, 2H), 6.57-6.63 (m, 2H), 7.04-7.31 (m, 12H), 7.41-7.46 (m, 2H); ^13^C NMR (75.5 MHz, CD_3_OD): δ 11.3, 16.0, 26.1, 30.4, 37.6, 37.9, 40.4, 43.3, 46.5, 56.2, 58.9, 59.3, 66.4, 70.2, 70.6, 101.7, 107.3, 115.4 (d, *J*_CF_ = 21.4 Hz, 2C), 124.4, 127.4, 129.4, 129.5, 130.1, 130.2, 132.0, 132.2 (d, *J*_CF_ = 8.1 Hz, 2C), 134.0, 136.1, 138.7, 140.6, 158.1, 160.9, 163.0 (d, *J*_CF_ = 243.9 Hz, 1C), 173.3, 177.6. HRMS calcd (M+H)^+^: 748.3529; found 748.3540. LC-MS Purity System A: *t*_R_ = 7.69 min, 98%; System B: *t*_R_ = 6.34 min, 96%.

#### (2R,4S,5S)-5-amino-2-benzyl-N-((2S,3S)-1-(4-chlorophenylamino)-3-methylpentan-2-yl)-6-(4'-fluoro-2-(3-methoxypropoxy)biphenyl-4-yloxy)-4-hydroxyhexanamide (28)

Compound **28** (colorless oil) was synthesized from **12c** according to the general procedures A-D using amine **P**. [α]_D_^22^ +10 (*c* 0.1, MeOH); ^1^H NMR (300 MHz, CD_3_OD): δ 0.84-0.92 (m, 6H), 1.08-1.20 (m, 1H), 1.45-1.53 (m, 1H), 1.58-1.66 (m, 1H), 1.69-1.74 (m, 1H), 1.92 (quintet, overlapped, *J* = 6.3 Hz, 2H), 1.88-1.98 (m, 1H), 2.72-2.91 (m, 4H), 2.95-3.01 (m, 2H), 3.26 (s, 3H), 3.42 (t, *J* = 6.3 Hz, 2H), 3.67-3.73 (m, 1H), 3.86-3.94 (m, 2H), 4.01 (t, overlapped, *J* = 6.3 Hz, 2H), 4.00-4.05 (m, 1H), 6.42-6.63 (m, 2H), 7.02-7.12 (m, 5H), 7.15-7.19 (m, 5H), 7.41-7.46 (m, 2H); ^13^C NMR (75.5 MHz, CD_3_OD): δ 11.8, 16.1, 26.1, 30.4, 37.4, 38.1, 40.6, 46.0, 47.1, 54.3, 56.3, 58.9, 66.4, 70.2, 70.3, 70.6, 101.7, 107.3, 114.9, 115.4 (d, *J*_CF_ = 21.2 Hz, 2C), 122.2, 124.4, 127.4, 129.4, 129.7, 130.1, 130.2, 132.0, 132.1 (d, *J*_CF_ = 8.0 Hz, 2C), 136.1, 140.8, 148.7, 158.1, 160.9, 163.1 (d, *J*_CF_ = 244.2 Hz, 1C), 177.6. HRMS calcd (M+H)^+^: 720.3580; found 720.3596. LC-MS Purity System A: *t*_R_ = 8.50 min, 100%; System B: *t*_R_ = 7.11 min, 96%. 

#### (2S,4S,5S)-5-amino-N-((2S,3S)-1-(4-chlorobenzylamino)-3-methyl-1-oxopentan-2-yl)-6-(4'-fluoro-2-(3-methoxyprop-oxy)biphenyl-4-yloxy)-4-hydroxy-2-isopropylhexanamide (29)

Compound **29** (colorless oil) was synthesized from **12d** according to the general procedures A-D. [α]_D_^22^ -12 (*c* 0.1, MeOH); ^1^H NMR (300 MHz, DMSO-d_6_): δ 0.75-0.82 (m, 9H), 0.87 (d, *J* =6.9 Hz, 3H), 1.08-1.17 (m, 1H), 1.21-1.25 (m, 1H), 1.40-1.48 (m, 1H), 1.60-1.78 (m, 3H), 1.82-1.90 (m, 2H), 2.21-2.31 (m, 1H), 2.78-2.83 (m, 1H), 3.20 (s, 3H), 3.37 (t, *J* = 6.3 Hz, 2H), 3.43-3.49 (m, 1H), 3.75-3.81 (m, 1H), 3.93 (dd, *J* = 6.9, 9.3 Hz, 1H), 4.00 (t, *J* = 6.3 Hz, 2H), 4.15-4.33 (m, 3H), 6.554-6.59 (m, 2H), 7.16-7.24 (m, 5H), 7.31-7.35 (m, 2H), 7.44-7.49 (m, 2H), 7.69 (d, *J* = 9.0 Hz, 1H), 8.49 (t, *J* = 6.0 Hz, 1H); ^13^C NMR (75.5 MHz, DMSO-d_6_): δ 10.6, 15.4, 20.2, 20.6, 24.4, 28.8, 30.5, 34.0, 36.1, 41.3, 49.0, 54.6, 56.7, 57.9, 65.0, 68.1, 68.4, 70.3, 100.2, 106.1, 114.6 (d, *J*_CF_ = 21.2 Hz, 2C), 121.4, 128.0, 129.0, 130.8, 130.9, 131.2, 134.3, 138.4, 156.2, 159.5, 160.9 (d, *J*_CF_ = 243.3 Hz, 1C), 171.4, 174.4. HRMS calcd (M+H)^+^: 700.3529; found 700.3547. LC-MS Purity System A: *t*_R_ = 7.27 min, 100%; System B: *t*_R_ = 5.79 min, 98%.

#### (2S,4S,5S)-5-amino-N-((2S,3S)-1-(4-chlorophenylamino)-3-methylpentan-2-yl)-6-(4'-fluoro-2-(3-methoxypropoxy)bi-phenyl-4-yloxy)-4-hydroxy-2-isopropylhexanamide (30)

Compound **30** (colorless oil) was synthesized from **12d** according to the general procedures A-D using amine **P**. [α]_D_^22^ -11 (*c* 0.1, MeOH); ^1^H NMR (300 MHz, DMSO-d_6_): δ 0.75-0.88 (m, 12H), 1.09-1.21 (m, 1H), 1.24-1.29 (m, 1H), 1.45-1.52 (m, 1H), 1.60-1.65 (m, 3H), 1.86 (quintet, *J* = 6.3 Hz, 2H), 2.17-2.24 (m, 1H), 2.81-2.85 (m, 1H), 2.97-3.15 (m, 2H), 3.19 (s, 3H), 3.37 (t, *J* = 6.3 Hz, 2H), 3.42-3.51 (m, 1H), 3.77-3.82 (m, 1H), 3.92-3.95 (m, 2H), 3.99 (t, *J* = 6.3 Hz, 2H), 5.46-5.50 (m, 1H), 6.50-6.62 (m, 4H), 7.05 (d, *J* = 2.2 Hz, 2H), 7.16-7.22 (m, 3H), 7.44-7.51 (m, 2H); ^13^C NMR (75.5 MHz, DMSO-d_6_): δ 11.3, 15.4, 20.2, 20.7, 24.5, 28.7, 30.3, 35.6, 40.9, 44.3, 49.5, 51.3, 54.8, 57.9, 65.0, 68.2, 68.4, 100.2, 106.1, 111.9, 113.2, 114.6 (d, *J*_CF_ = 21.2 Hz, 2C), 118.7, 121.5, 128.5, 130.8, 130.9, 134.3, 147.6, 156.2, 159.4, 162.5, 174.6. HRMS calcd (M+H)^+^: 672.3580; found 672.3593. LC-MS Purity System A: *t*_R_ = 8.04 min, 98%; System B: *t*_R_ = 5.88 min, 96%.

## Figures and Tables

**Fig. (1):**
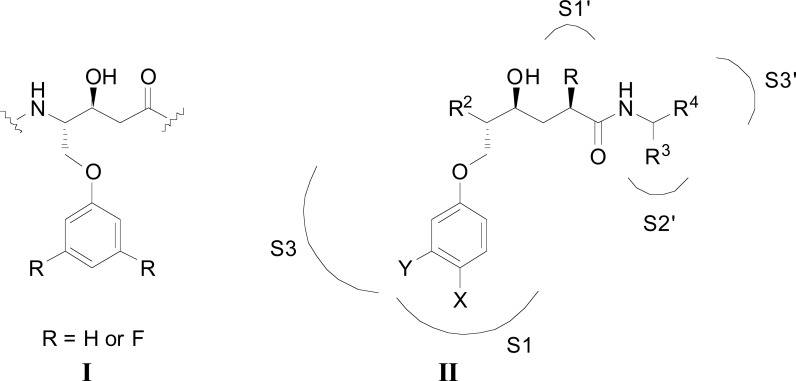
A statine-based central core (**I**) described in a previous report on potent BACE-1 inhibitors [7]; and a general illustration of the hydroxyethylene (HE) isostere (**II**) (with R, X and Y substituents) that was used in this work. Different P1´ residues were incorporated into the HE isostere with the aim to find improved inhibitor interactions. The purpose of the X and Y substituents on the P1 residue was to protrude deeper into the S1 pocket and to extend into the S3 pocket, respectively.

**Fig. (2):**
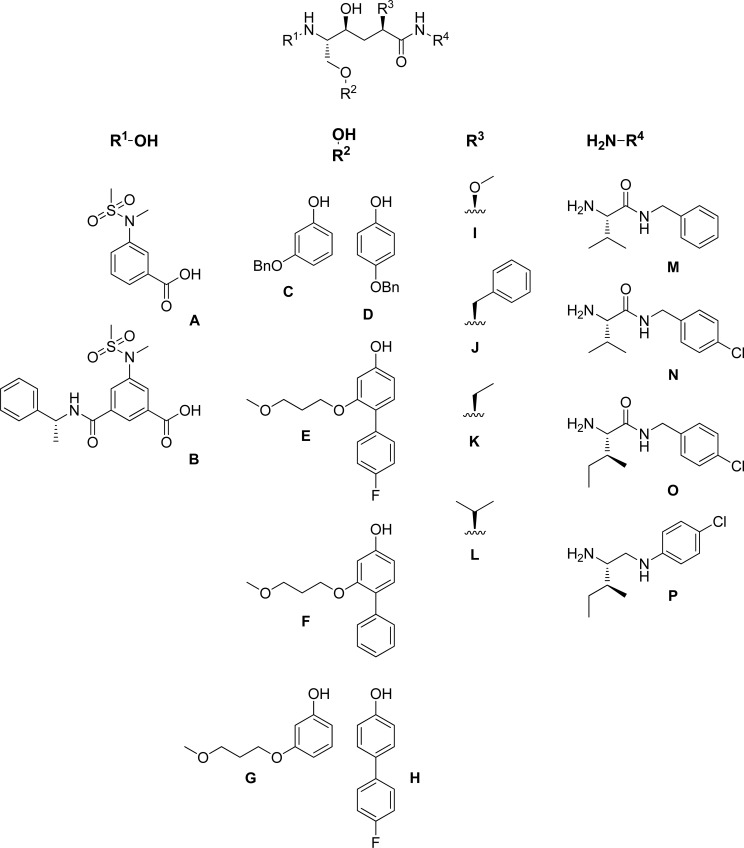
A general illustration of the hydroxyethylene central core and the different R substituents that were used.

**Table 1:** Target compounds and inhibition data.

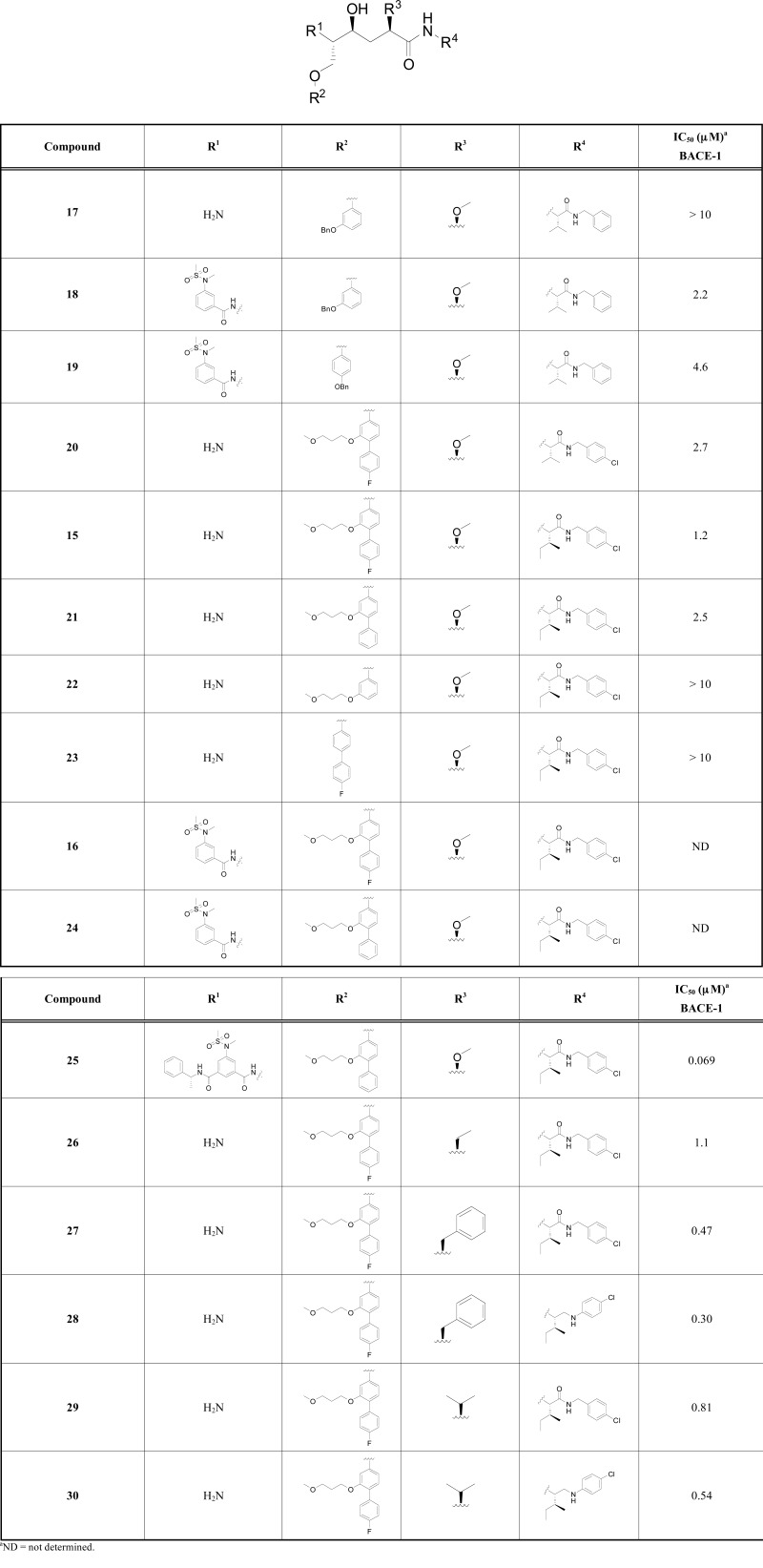

## References

[1] Melnikova I (2007). Therapies for Alzheimer's disease. Nat. Rev. Drug Discov.

[2] Hardy J, Selkoe D J (2002). The amyloid hypothesis of Alzheimer's disease: progress and problems on the road to therapeutics. Science.

[3] Luo Y, Bolon B, Kahn S, Bennett B D, Babu-Khan S, Denis P, Fan W, Kha H, Zhang J, Gong Y, Martin L, Louis J C, Yan Q, Richards W G, Citron M, Vassar R (2001). Mice deficient in BACE1, the Alzheimer's beta-secretase, have normal phenotype and abolished beta-amyloid generation. Nat. Neurosci.

[4] Roberds S L, Anderson J, Basi G, Bienkowski M J, Branstetter D G, Chen K S, Freedman S B, Frigon N L, Games D, Hu K, Johnson-Wood K, Kappenman K E, Kawabe T T, Kola I, Kuehn R, Lee M, Liu W, Motter R, Nichols N F, Power M, Robertson D W, Schenk D, Schoor M, Shopp G M, Shuck M E, Sinha S, Svensson K A, Tatsuno G, Tintrup H, Wijsman J, Wright S, McConlogue L (2001). BACE knockout mice are healthy despite lacking the primary beta-secretase activity in brain implications for Alzheimer's disease therapeutics. Hum. Mol. Genet.

[5] Johansson P O, Lindberg J, Blackman M J, Kvarnström I, Vrang L, Hamelink E, Hallberg A, Rosenquist, Samuelsson B (2005). Design and synthesis of potent inhibitors of plasmepsin I and II: X-ray crystal structure of inhibitor in complex with plasmepsin II. J. Med. Chem.

[6] Rahuel J, Rasetti V, Maibaum J, Rueger H, Goschke R, Cohen N C, Stutz S, Cumin F, Fuhrer W, Wood J M, Grutter M G (2000). Structure-based drug design: the discovery of novel non-peptide orally active inhibitors of human renin. Chem. Biol.

[7] Bäck M, Nyhlén J, Kvarnström I, Appelgren S, Borkakoti N, Jansson K, Lindberg J, Nyström S, Hallberg A, Rosenquist, Samuelsson B (2008). Design, synthesis and SAR of potent statine-based BACE-1 inhibitors: exploration of P1 phenoxy and benzyloxy residues. Bioorg. Med. Chem.

[8] Björklund C, Oscarson S, Benkestock K, Borkakoti N, Jansson K, Lindberg J, Vrang L, Hallberg A, Rosenquist, Samuelsson B (2010). Design and synthesis of potent and selective BACE-1 inhibitors. J. Med. Chem.

[9] Simas A B C, Coelho A L, Costa P R R (1999). Regioselective Lithiation of Resorcinol Derivatives: Synthesis of Mono O-MOM- and O-Benzylresorcinols Prenylated at C-2 or C-4. Positions Synthesis-Stuttgart.

[10] Barber C G, Bunnage M E, Harvey J W, Mathias J P (2005). Compounds. U.S. Patent 2005/0020611.

[11] Stachel S J, Coburn C A, Steele T G, Jones K G, Loutzenhiser E F, Gregro A R, Rajapakse H A, Lai M T, Crouthamel M C, Xu M, Tugusheva K, Lineberger J E, Pietrak B L, Espeseth A S, Shi X P, Chen-Dodson E, Holloway M K, Munshi S, Simon A J, Kuo L, Vacca J P (2004). Structure-based design of potent and selective cell-permeable inhibitors of human beta-secretase (BACE-1). J. Med. Chem.

[12] Arslan T, Abraham A T, Hecht S M (1998). Structurally altered substrates for DNA topoisomerase I. Effects of inclusion of a single 3'-deoxynucleotide within the scissile strand. Nucleos. Nucleot.

[13] Wångsell F, Gustafsson K, Kvarnström I, Borkakoti N, Edlund M, Jansson K, Lindberg J, Hallberg A, Rosenquist, Samuelsson B (2010). Synthesis of potent BACE-1 inhibitors incorporating a hydroxyethylene isostere as central core. Eur. J. Med. Chem.

[14] Rasmussen J R, Slinger C J, Kordish R J, Newman-Evans D D (1981). Synthesis of deoxy sugars. Deoxygenation by treatment with N,N'-thiocarbonyldiimidazole/tri-n-butylstannane. J. Org. Chem.

[15] Rondot B, Durand T, Girard J P, Rossi J C, Schio L, Khanapure S P, Rokach J (1993). A Free-Radical Route to Syn Lactones and Other Prostanoid Intermediates in Isoprostaglandin Synthesis. Tet. Lett.

[16] Muller M, Huchel U, Geyer A, Schmidt R R (1999). Efficient intramolecular glycosylation supported by a rigid spacer. J. Org. Chem.

[17] Crich D, Vinogradova O (2007). Facile oxidative cleavage of 4-O-benzyl ethers with dichlorodicyanoquinone in rhamno- and mannopyranosides. J. Org. Chem.

[18] Tagad H D, Hamada Y, Nguyen J T, Hidaka K, Hamada T, Sohma Y, Kimura T, Kiso Y (2011). Structure-guided design and synthesis of P1' position 1-phenylcycloalkylamine-derived pentapeptidic BACE1 inhibitors. Bioorg. Med. Chem.

